# Source, transport, and fate of nitrate in shallow groundwater in the eastern Niger Delta

**DOI:** 10.1007/s11356-024-35499-6

**Published:** 2024-11-20

**Authors:** Dogo Lawrence Aleku, Kirstin Dähnke, Thomas Pichler

**Affiliations:** 1 Institute of Geosciences, University of Bremen, 28359 Bremen, Germany; 2 Institute for Carbon Cycles, Helmholtz-Zentrum Hereon, 21502 Geesthacht, Germany

**Keywords:** Nitrate, Nitrite, Groundwater, Source, Sewage, Isotopic, Hydrochemical, Contamination, Anthropogenic

## Abstract

The eastern Niger Delta region in Nigeria is a hotspot for reactive nitrogen pollution due to extensive animal husbandry, pit latrine usage, and agricultural practices. Despite the high level of human activity, the sources and processes affecting nitrogen in groundwater remain understudied. Groundwater nitrate (NO_3_^−^) concentrations are highly variable, with some areas recording values well above the safe drinking water threshold of 50 mg/L. This is particularly true near municipal sewage systems. Elevated nitrite (NO_2_^−^) and ammonium (NH_4_^+^) concentrations were also detected in the study area. Sewage analysis revealed NO_3_^−^ concentrations ranging from 1 to 145 mg/L, NO_2_^−^ from 0.2 to 2 mg/L, and notably high NH_4_^+^ concentrations. A comparison of major ions indicated that 71%, 90%, 87%, and 92% of groundwater samples surpassed reference site levels for calcium (Ca^2+^), sodium (Na^+^), potassium (K^+^), and chloride (Cl^−^), respectively, pointing to sewage as a likely source of contamination. The NO_3_^−^/Cl^−^ ratios at several sites suggested that most groundwater NO_3_^−^ originates from human waste. Stable isotope analysis of NO_3_^−^ showed a general enrichment in ^15^N and, in some cases, a depletion in ^18^O, indicating that the NO_3_^−^ originates from sewage-derived NH_4_^+^ nitrification. Although denitrification, a process that reduces NO_3_^−^, is present, the high dissolved oxygen (DO) and NO_3_^−^ levels in the groundwater suggest that denitrification is insufficient to fully mitigate NO_3_^−^ pollution. Consequently, there is a risk of NO_3_^−^ leaching from shallow aquifers into the Niger Delta’s surface waters and ultimately into the coastal ocean.

**Supplementary Information:**

The online version contains supplementary material available at 10.1007/s11356-024-35499-6.

## Introduction

Globally, excess nitrate (NO_3_^−^) in groundwater is an environmental problem threatening human health, either directly due to its adverse health effects or by inducing the release of toxic metals, such as cadmium (Cd) from the aquifer matrix (e.g., Kubier et al. 2020, 2019; Ward et al. 2018). Migration of NO_3_^−^ from groundwater to surface waters and subsequently into the coastal ocean has also become a cause of concern worldwide (e.g., Guo et al. 2021; Harris et al. 2022). As a result, various global organizations have implemented measures to reduce NO_3_^−^ levels in groundwater. For instance, the European Union (EU) established a range of measures to reduce NO_3_^−^ contributions from agricultural and non-agricultural sources in the EU (Stark & Richards 2008). To this effect, the EU selected a concentration of 50 mg/L as the guideline value for NO_3_^−^ in groundwater. Similarly, several countries, including Nigeria (NSDWQ 2015), and organizations like the World Health Organization (WHO 2017) have set 50 mg/L as the guideline for NO_3_^−^ in drinking water.

Various authors investigated the groundwater quality and geochemistry in Nigeria's urban and rural areas (Abanyie et al. 2023; Eludoyin and Fajiwe 2023; Obrike et al. 2022; Raimi et al. 2023). Some studies included NO_3_^−^ and concentrations reported were up to 2.1 mg/L in Nnewi, 4.2 mg/L in Awka (Ayejoto & Egbueri 2023), 21.1 mg/L in Umunya (Egbueri et al. 2023), 36 mg/L in Ogbaru (Unigwe et al. 2022), 157 mg/L in Gboko (Omonona & Okogbue 2021), and up to 770 mg/L in Maiduguri (Goni et al. 2019). NO_3_^−^ sources were not identified in those studies, although agricultural activities, pit latrines, and animal waste were hypothesized as possible sources. Similar investigations in other parts of the world suggested that, most commonly, anthropogenic sources such as nitrogen fertilizer, manure, municipal and domestic sewage discharge, pit latrines, soil organic nitrogen, and atmospheric deposition contribute to NO_3_^−^ loading of groundwaters (Biddau et al. 2023; Kendall et al. 2007).

To effectively reduce the excess levels of NO_3_^−^ in groundwater, improved groundwater management practices that minimize the release of nitrogen compounds into the environment are required. Determining NO_3_^−^ sources and variability is essential to improve nitrogen management practices. However, identifying a given source and its contribution can be complicated when multiple nitrogen sources exist. Such uncertainty, for instance, is typical in urban areas where intensive agricultural activities involving nitrogen fertilizers are common (Minet et al. 2017). Hence, accurately identifying the NO_3_^−^ source(s) in groundwater, evaluating the ongoing biogeochemical process in the aquifer, and calculating NO_3_^−^ contributions from different potential sources are necessary for effective management measures to reduce NO_3_^−^ levels in groundwater.

Stable oxygen and nitrogen isotopic signatures of NO_3_^−^ have been effectively applied to identify NO_3_^−^ sources while also detecting nitrification, denitrification, or dilution in groundwater (e.g., Anornu et al. 2017; Carrey et al. 2021; Guo et al. 2020; Harris et al. 2022). However, uncertainties remain during data interpretation, which include (1) significant overlaps resulting from multiple nitrogen sources during the early leaching process within unsaturated zones or as nitrification proceeds and (2) a mixing process between the multiple nitrogen sources, subsequent NO_3_^−^ removal due to denitrification (Kendall et al. 2007), and the concurrent productions of NO_3_^−^ during anaerobic ammonium oxidation under limited oxygen conditions (Granger & Wankel 2016). This complicates nitrogen source identification in groundwaters (Kendall et al. 2007); hence, the need for an approach that combines major ion and isotope data to reduce such uncertainties (Minet et al. 2017). This is possible because, for instance, municipal sewage and animal wastes are typically enriched with chloride (Cl^−^), potassium (K^+^), and sodium (Na^+^), among many other contaminants, which are all released by decomposing organic matter (e.g., Ranjbar & Jalali 2012).

With this in mind, we combined stable NO_3_^−^ isotope data with hydrochemical markers (i.e., Ca^2+^, Na^+^, K^+^, and Cl^−^) for nitrogen source identification. It is important to note that there are no available studies on groundwater NO_3_^−^ in the eastern Niger Delta, despite the widespread nitrogen-related anthropogenic activities. Hence, this study presents a unique opportunity to investigate NO_3_^−^ and NO_2_^−^ source, transport, and fate across the eastern Niger Delta groundwater systems to improve management and remediation efforts.

## Materials and methods

### Site description, geology, and hydrogeology

The study site is in the eastern Niger Delta Region of Nigeria (latitude 4°44ʹ57″N to 4°47ʹ42″N and longitude 7°05ʹ26″E to 7°09ʹ54″E) and comprises the following communities: Alesa, Ogale, Ebubu, Alode and Okochiri, with an uneven topography which varied between 0.1 and 64.5 m above sea level. The area has two distinct seasons — the wet (March to October) and dry (November to February) seasons. The mean monthly temperature is high in March/April (up to 26.7 °C) and low in July/August (24.4 °C). Humidity ranges from 60 to 90% and is associated with warm and dry northeastern winds (Hassan et al. 2020). Mean annual precipitation widely varies between 2800 and 4000 mm/year (Ohwoghere-Asuma et al. 2023).

**Fig. 1:**
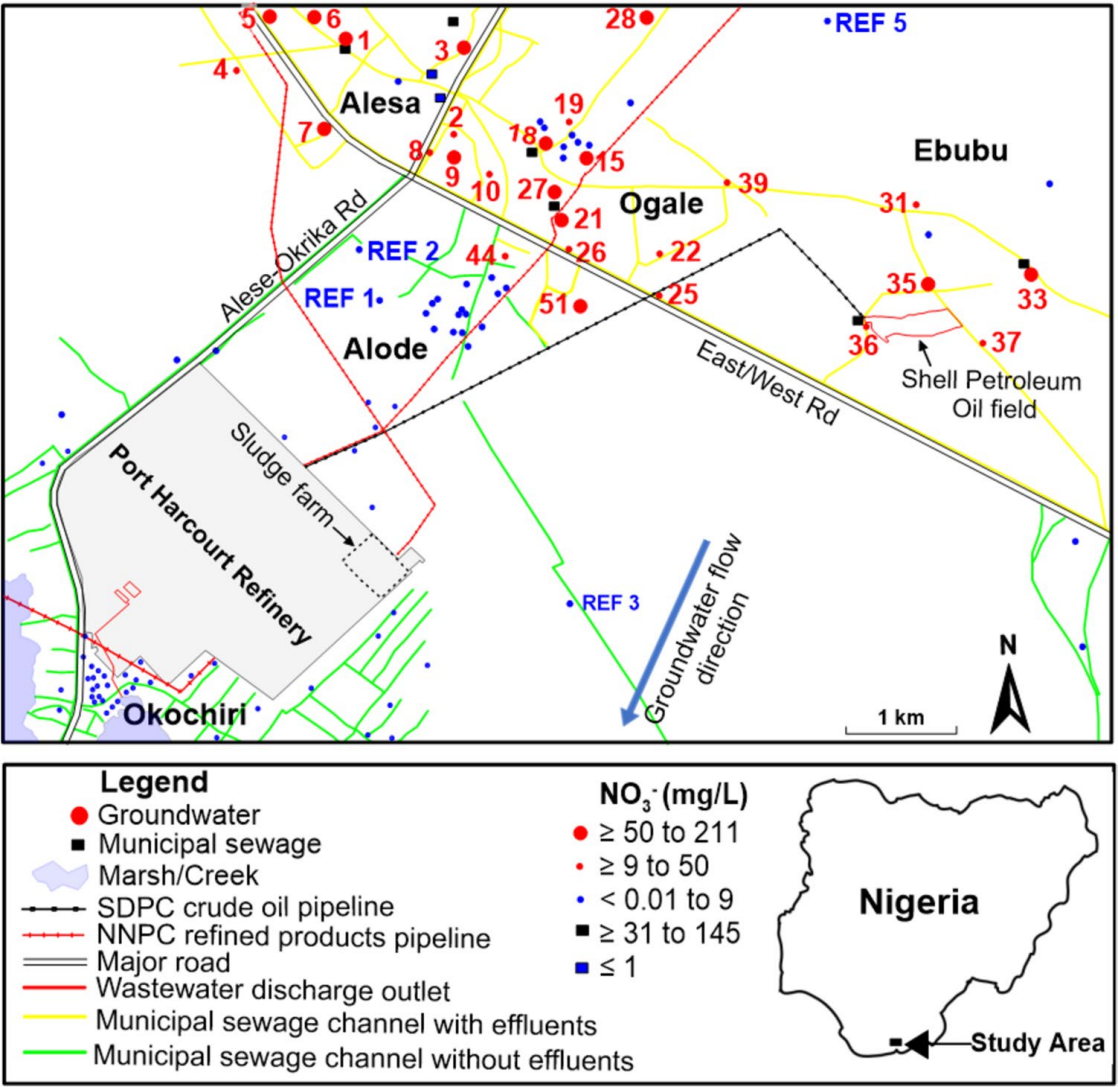
The spatial distribution of NO_3_^−^ concentrations in the groundwater and municipal sewage across the study area. Only samples with elevated NO_3_^−^ concentrations were labeled on the map for clarity. For color interpretation in this map, the reader is referred to the web version of this article

The sampling locations are shown in Fig. 1. Alesa, Ogale, and Ebubu are in the northern part of the study area, commonly characterized by the presence of (1) municipal and domestic sewage in the drainage systems and (2) unlined pit latrine toilets for human excrement. In these communities, the sewage flow was hindered by blockage resulting from indiscriminate solid waste disposal and the gentle nature of the topography (Fig. S1). The municipal sewage was more commonly observed in Alesa than in Ogale and Ebubu. In contrast, sewage was not observed in the Alode and Okochiri drainage systems. The steep nature of the topography appears to play an essential role in aiding the free flow and eventual absence of municipal sewage (Fig. S2).

Three major lithostratigraphic units have been identified within the Niger Delta Basin: the Benin Formation, Agbada Formation, and Akata Formation (Obaje 2009). The Oligocene to Recent Benin Formation is about 2 km thick and predominantly consists of clay units, coarse-grained, sub-angular to well-rounded, poorly sorted coastal plain sand and alluvial deposits of about 95 to 99% quartz grains at shallow depths (Nwajide 2013). The formation serves as a groundwater reservoir for the region (Adelana 2008). The aquifer is recharged mainly by direct precipitation and exfiltration from major regional rivers (Abam & Nwankwoala 2020). The sandy and permeable nature of the aquifer further facilitates rapid infiltration into the upper units of the formation (Abam & Nwankwoala 2020). However, the anthropogenic activities in the region have left the shallow groundwater vulnerable to pollution (Adeniran et al. 2023).

### Groundwater sampling

The groundwater samples for this study were collected from shallow wells (1 to 30 m) in the Benin Formation in April 2022 and April 2023. Groundwater and sewage samples were collected from communities with municipal and domestic sewage and areas considered relatively unaffected by municipal wastewater (i.e., reference sites 1 to 5). The reference samples were collected from Alode (Refs 1 and 2), Okochiri (Ref 3), Okrika Island (Ref 4), and Ogale (Ref 5) within the same geological unit in relatively new residential areas without municipal wastewater or other potential anthropogenic contamination sources.

The groundwater samples were collected either (1) manually, using a water bailer made of polyvinyl chloride, or (2) with an electric submersible pump in cases where those were installed in the wells. First, groundwater was pumped into the overhead storage tank to purge the wells for 30 min before sampling directly from the wellhead. The bailer was rinsed three times with the groundwater before sampling. Sampling was conducted during the early hours (between 6:00 and 8:00 a.m.) when the wells were actively used to ensure that fresh samples were collected. However, for most of the wells, water table and well depth measurements were not possible because well heads were sealed with concrete slabs (Fig. S3) to protect wells from surface contamination and theft of submersible pumps. The well owners rejected unsealing the wells for depth measurement. Nevertheless, in those wells where measurement was possible, the water table varied between 1.5 and 9 m, whereas well depths ranged from 9.8 to 30 m. In total, 180 samples (105 in 2022 and 75 in 2023) were collected from private supply wells (PSW) and community supply wells (CSW) next to municipal or domestic sewage drainages. In private residences, most wells were next to pit latrine toilets, usually between 2 and 9 m apart.

Immediately after collection, the samples were filtered through 0.45 μm cellulose acetate (CA) membranes and separated into aliquots for the different chemical analyses (isotopes, major ions, and dissolved organic carbon). The samples were stored in 25-mL glass vials for DOC, 30 mL brown HDPE vials for major cations, and 20 mL clear HDPE vials for anions and isotopes. The sub-samples for DOC and major cations were preserved with 2% concentrated nitric acid (HNO_3_). All samples were stored at 4 °C until laboratory analyses.

The pH, conductivity (EC), total dissolved solids (TDS), temperature, dissolved oxygen (DO), salinity, redox potential (ORP), and resistivity were determined immediately in situ using a Hanna instrument HI98494 multiparameter. In the field, the total alkalinity (CaCO_3_) was determined by colorimetric titration with 0.16 N H_2_SO_4_ in combination with a bromcresol green-methyl red indicator. The bromcresol green-methyl red indicator powder was added to 100 mL of the groundwater sample and titrated using a Hach digital titrator to a light pink color. The total alkalinity was reported as mg/L CaCO_3_.

Additionally, eight samples were collected from municipal and domestic sewage in Alesa, Ogale, and Ebubu. The samples were filtered through 0.45-μm cellulose acetate (CA) membrane filters and collected into 20-mL clear HDPE vials.

### Analytical procedures

#### Cation, anion, and DOC measurements

Major cations and trace elements were determined by inductively coupled plasma-optical emission spectrometry (ICP-OES) using a PerkinElmer Optima 7300 DV instrument. The precision of the measurement was checked using EnviroMAT Groundwater Low (ES-L-2) and High (ES-H-2) certified water from SCP Science, Canada, showing errors of < 3% for all analytes. Major anions (including NO_3_^−^ and NO_2_^−^) were determined less than 28 days after sampling using a Metrohm 883 Basic IC plus instrument with a 5-μL injection loop and a Metrosep A Supp5 (150 × 4.0 mm; 5 μm) column. An internal standard was used to check the accuracy and precision of the measurement, and errors of less than 10% were recorded.

Dissolved organic carbon (DOC), the fraction of organic carbon that can pass through a 0.45-μm pore size, was determined using a Shimadzu TOC analyzer TOC-V CPN (Shimadzu Corporation). A certified total organic carbon standard of 50 mg/L (Aqua Solutions) was used for quality control, and the measurement error was determined to be less than 6%.

The ammonium (NH_4_^+^) was determined photometrically at 655 nm with salicylate following standard procedures (DIN 38406, 1983). Determination was carried out approximately 8 months after sampling; thus, some microbial conversion could have occurred. Although filtration should have at least partly sterilized the sample.

### Determination of NO^3−^ isotopes (δ^15^N-NO^3−^ and δ^18^O-NO^3.−^)

A subset of 20 groundwater and 2 municipal wastewater samples were analyzed for stable isotopes, specifically from wells where their owners had granted permission to collect samples. The ^15^N/^14^N and ^18^O/^16^O ratios in dissolved NO_3_^−^ were measured and expressed as δ^15^N-NO_3_^−^ and δ^18^O-NO_3_^−^. Isotope ratios were determined following the denitrifier method (Casciotti et al. 2002; Sigman et al. 2001). NO_3_^−^ and NO_2_^−^ are quantitatively converted to nitrous oxide (N_2_O) by the denitrifying bacteria (*Pseudomonas aureofaciens*, ATCC#13,985) that lack N_2_O reductase. The sample volume for isotope determination was adjusted to achieve 10 nmol of N_2_O. N_2_O was extracted from the sample vials by purging with helium and measured with a GasBench II (Thermo, Germany), coupled to an isotope ratio mass spectrometer (Delta Plus XP, Thermo, Germany). For quality assurance, two external standards (USGS34: δ^15^N − 1.8‰, δ^18^O − 27.9‰; IAEA − NO_3_^−^: δ^15^N + 4.7‰, δ^18^O + 25.6‰) and one internal standard were measured with each sample batch. The standard deviation of samples and standards was < 0.2‰ for δ ^15^N-NO_3_^−^ (*n* = 4) and < 0.5‰ for δ ^18^O-NO_3_^−^ (*n* = 4). Note that this method yields combined isotope values for NO_3_^−^  + NO_2_^−^. In two samples, NO_2_^−^ concentration exceeded 5% of the nitrate concentration. These samples were excluded from the isotopic analysis.

## Results

### Groundwater hydrochemical characteristics

The supplemental information (SI) Tables 1S and 2S present data for all samples, including minimum, maximum, median and average. The pH ranged from 3.5 to 6.9, temperature from 25 to 34 °C, EC from 16 to 852 µS/cm, TDS from 8 to 427 mg/L, DO from 0.7 to 8.9 mg/L, and salinity from 0.01 to 0.4 PSU. The EC ranged from 17 to 69 µS/cm at the reference site, and the TDS ranged from 9 to 32 mg/L.

Most groundwater quality parameters at the contaminated and reference sites were in accordance with WHO (2017) guidelines for drinking water. Nevertheless, the parameters associated with contamination from NO_3_^−^ fertilizer and animal/human waste effluents (Cl^−^ and K^+^) or animal/human wastes (Na^+^) (Minet et al. 2017) showed higher concentrations at the contaminated sites than those at the reference sites. The concentrations of Na^+^ in groundwater ranged from 1 to 56 mg/L, and 57% of the samples exceeded the measured reference value range of 1 to 2 mg/L. The concentrations of K^+^ ranged from 0.1 to 59 mg/L, and 33% of the samples exceeded the 0.3 to 0.6 mg/L range at the reference sites. The concentrations of Cl^−^ ranged from 1 to 66 mg/L, with 25% of the samples exceeding the 2 to 5-mg/L range at the reference site. Ca^2+^ ranged from 0.2 to 51 mg/L, and 71% of the samples exceeded the 1-mg/L range at the reference sites. In the sewage, Na levels ranged from 5 to 363 mg/L, K^+^ concentration from 1 to 74 mg/L, Cl^−^ concentrations from 28 to 242 mg/L, and Ca^2+^ concentrations from 22 to 65 mg/L. Generally, 90%, 87%, and 92% of the groundwater samples exceeded the Na^+^, K^+^, and Cl^−^ concentrations measured at the reference sites, respectively.

Similarly, although the Mg^2+^, F^−^, and SO_4_^2−^ levels were relatively low in the groundwater and sewage, their concentrations exceeded the reference site values in several samples (Tables 2S and 3S). Here, the Mg^2+^, F^−^, and SO_4_^2−^ levels showed that 42%, 34%, and 18% of the groundwater samples exceeded their respective reference site concentrations. NO_3_^−^ vs Mg had a strong positive correlation (*R* = 0.9), likewise NO_3_^−^ vs F^−^ (*R* = 0.53), and NO_3_^−^ vs SO_4_^2−^ (*R* = 0.64). Those elevated ion levels also indicated a possible anthropogenic influence, most likely sewage infiltration into the aquifer.

**Fig. 2:**
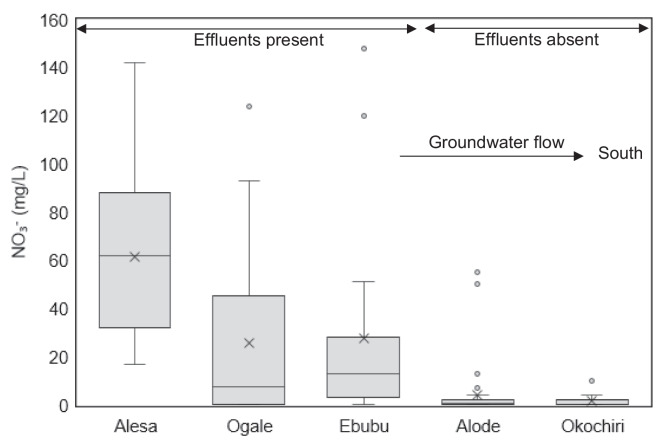
Box plot of NO_3_^−^ concentrations in the study area. Alesa, Ogale, and Ebubu are in the northern part of the study site, Alode is in the central part, whereas Okochiri is in the south. An outlier (211 mg/L) in Ogale was excluded from the plot. The edges of the box represent the 75th and 25th percentiles, respectively. The “x” sign in the box represents the mean value. The solid line represents the median value. The branch gives the range of the data except for the outliers. Twenty samples were selected across Alesa, Ogale, and Ebubu for the δ^15^N-NO_3_^−^ and δ^18^O-NO_3_^−^ analyses

The concentration of dissolved NO_3_^−^ in the water samples ranged from less than 0.01 up to 211 mg/L. Out of the 180 samples collected, 24 had concentrations that exceeded the maximum guideline value of 50 mg/L for NO_3_^−^ in drinking water. Elevated concentrations were observed in 2022 and 2023 in Alesa, Ogale, and Ebubu. In general, the groundwater NO_3_^−^ concentrations in groundwater were higher in the northern part of the study area (Alesa, Ogale, and Ebubu), where municipal sewage was frequently present. In contrast, in the southern part (Alode and Okochiri), where municipal sewage was absent, concentrations were comparably lower, not reaching the WHO guidelines (Fig. 2). In the sewage samples, NO_3_^−^ concentrations, up to 145, 131, and 100 mg/L, were detected in Alesa, Ogale, and Ebubu, respectively.

Eleven groundwater samples had nitrite (NO_2_^−^) concentrations that exceeded the NSDWQ (2015) drinking water guideline value of 0.2 mg/L. Concentrations up to 1 mg/L, 0.2 mg/L, 1 mg/L, 2 mg/L, and 0.2 mg/L were detected in Alesa, Ogale, Ebubu, Alode, and Okochiri groundwaters, respectively.

Ammonia (NH_4_^+^) was detected in five samples of the Alesa groundwater. The estimated concentration ranged from 0.02 to 1.6 mg/L. In Ogale, NH_4_^+^ was detected in all the samples, with concentration estimates ranging from < 0.02 to 12.7 mg/L. In Ebubu, however, NH_4_ was detected in only one sample (0.6 mg/L). Additionally, two sewage samples from Alesa were examined: EF 5 contained an estimated 4.8 mg/L, while NH_4_^+^ in EF 2 exceeded the instrument’s detection limit. Ammonia might have been lost due to prolonged storage. Hence, those values were considered minimum concentrations and only considered qualitatively.

### Hydrochemical facies

**Fig. 3:**
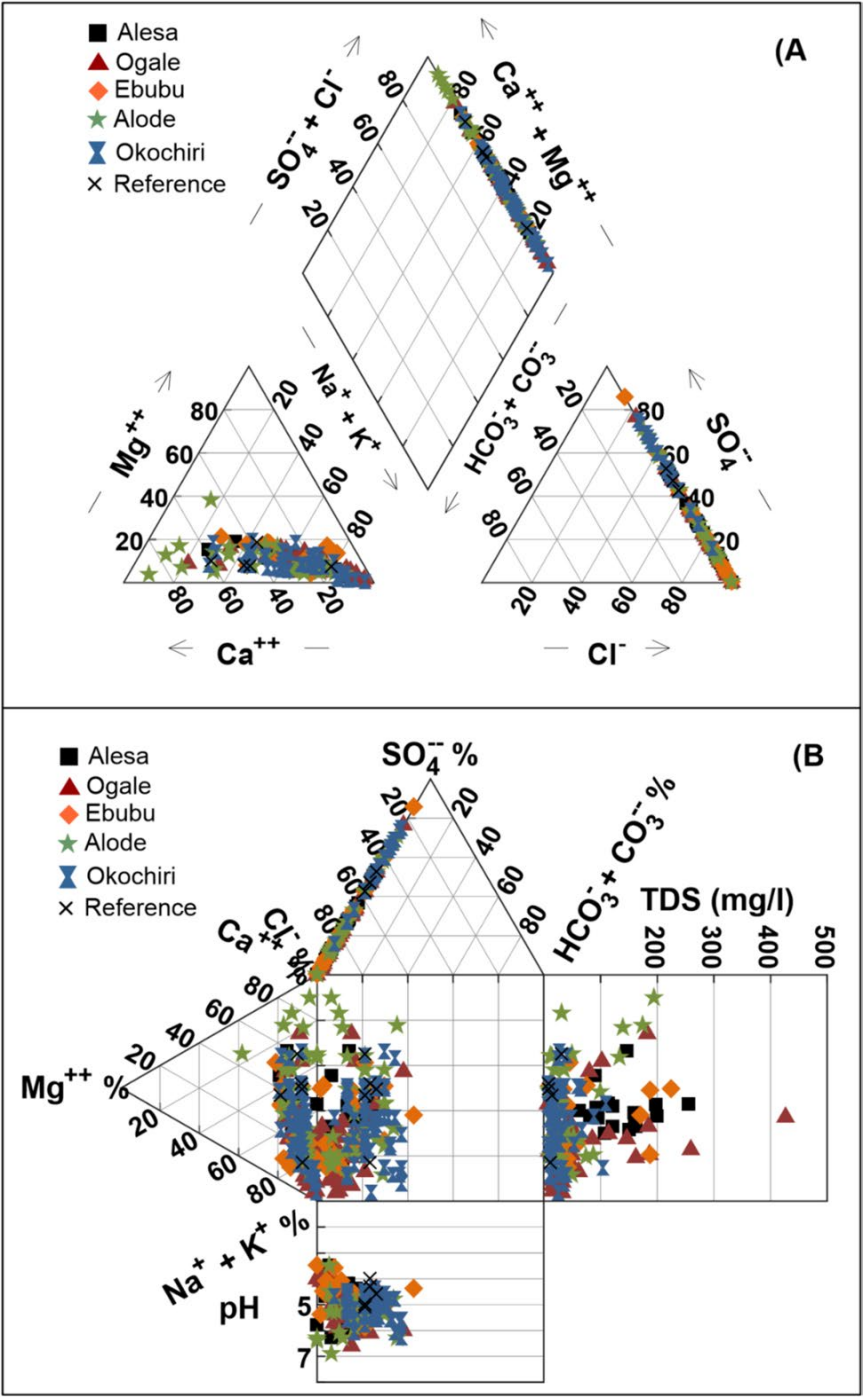
(**A**) Piper and (**B**) Durov diagrams for groundwater of the study area

Based on the major ions, the hydrochemistry of the groundwater was evaluated through the trilinear Piper (Piper 1944), Druov (Durov 1948), and Stiff diagrams using the Geochemist’s Workbench 17.0.2. Based on the Piper diagram given in Fig. 3a, two water types were identified from the groundwater samples: the Ca–Cl (23%) and Na-Cl (77%) water types. In the Alesa, the facies are 25% Ca–Cl and 75% Na-Cl type. In the Ogale, the facies are 10% Ca–Cl and 90% Na-Cl type, whereas in the Ebubu, the facies are 50% Ca–Cl and 50% Na-Cl type. Similarly, in the trilinear Durov diagram shown in Fig. 3b, all the groundwater samples have TDS less than 500 mg/L. Cl^−^ and SO_4_^2−^ are the dominant anions, and Na and Ca are the dominant cations. Furthermore, the Durov plot indicates the possible occurrence of NO_3_^−^, Cl^−^, and SO_4_^2−^ contamination in the investigated groundwater. As indicated by the Piper diagram, the Durov diagram also showed Na-Cl as the dominant water type in the study area. However, based on our salinity data, there was no incidence of saltwater intrusion in the groundwater despite the coastal nature of the area**.** The salinity values in the groundwater ranged from 0.04 to 0.2 (Alesa), 0.01 to 0.4 (Ogale), 0.01 to 0.2 (Ebubu and Alode), and 0.01 to 0.1 (Okochiri) PSU. Therefore, anthropogenic influence, rather than the mixing of freshwater with saltwater, was responsible for the occurrence of the Na-Cl water type in this study. All the groundwater samples in the study area showed freshwater facies. Notably, the elevated NO_3_^−^ values were not specific to a particular water type; nevertheless, values of each major ion were higher in the NO_3_^−^ contaminated sites (i.e., Alesa, Ogale, and Ebubu) and lower in the uncontaminated sites (i.e., Alode and Okochiri) as shown in the Stiff diagram in Fig. S4. Generally, the abundance of the anions and cations in the groundwater followed the order of Cl^−^  > SO_4_^2−^  > F^−^ and Na^+^  > K^+^  > Ca^2+^  > Mg^2+^, respectively.

### δ^15^N-NO_3_^−^ and δ^18^O-NO_3_^−^

**Fig. 4:**
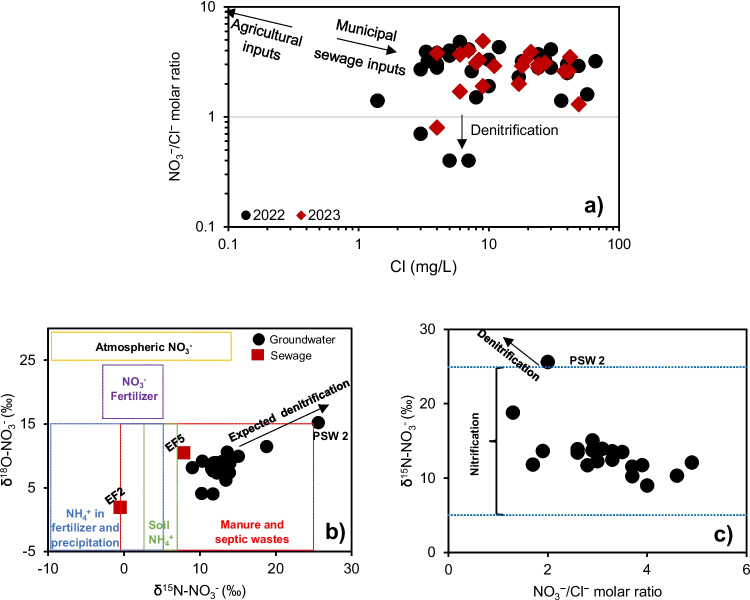
**a** Relationship between Cl^−^ concentrations vs NO_3_^−^/Cl^−^ molar ratios. **b** A plot of δ^15^N-NO_3_^−^ vs δ^18^O-NO_3_^−^ isotopic values in Alesa, Ogale, and Ebubu groundwater and sewage. The diagram was modified from Kendall et al. (2007), showing typical values of δ^15^N-NO_3_^−^ and δ^18^O-NO_3_^−^ derived or nitrified from leachates originating from the municipal sewage and pit latrine systems. The arrow shows the expected denitrification of NO_3_^−^ in the area. **c** A plot of δ^15^N-NO_3_^−^ vs NO_3_^−^/Cl^−^ molar ratio variation in the Alesa, Ogale, and Ebubu groundwater

In the groundwater, the δ^15^N-NO_3_^−^ isotopic signatures varied between + 8.9 and + 25.6‰, and δ^18^O-NO_3_^−^ varied between + 4.0 and + 15.2‰ (Fig. 4b; Table 1). Overall, the variation in the δ^15^N-NO_3_^−^ and δ^18^O-NO_3_^−^ values across Alesa, Ogale, Ebubu, and Alode was small (Table 1). The δ^18^O-NO_3_^−^ values tended to increase with the δ^15^N-NO_3_^−^ values for the groundwater samples collected in Alesa and Ogale, while this trend was not observed in Ebubu. The δ^15^N-NO_3_^−^ and δ^18^O-NO_3_^−^ values in the shallow groundwater fitted the regression lines for Alesa and Ogale (*y* = 0.58*x* + 0.21, *r*^2^ = 0.71).

**Table 1:** δ^15^N, δ^18^O, and NH_4_ values at each of the target communities

ID	Site	δ^15^N (‰)	δ^18^O (‰)	δ^15^N / δ^18^O	NH_4_ (µmol/L) estimates	NO₃^−^ (mg/L)	Cl^−^ (mg/L)	NO₃^−^/Cl^−^	Ln (NO_3_^−^)
1	Alesa	13.4	6.2	2.17	N.D	106	41	2.6	4.66
2	Alesa	25.6	15.2	1.68	B.D.L	34	17	2	3.53
3	Alesa	13.9	8.8	1.58	N.D	83	27	3.1	4.42
4	Alesa	13.6	9.4	1.45	0.03	17	9	1.9	2.83
5	Alesa	12.3	7.3	1.68	1.6	73	24	3	4.29
6	Alesa	13.7	7.8	1.76	B.D.L	53	18	2.9	3.97
7	Alesa	13.9	7.4	1.89	1.7	97	38	2.6	4.57
8	Alesa	15.1	9.9	1.52	0.4	32	11	2.9	3.47
9	Alesa	11.7	4	2.92	1.5	66	24	2.8	4.19
10	Alesa	10.2	4.1	2.5	N.D	22	6	3.7	3.09
15	Ogale	18.8	11.5	1.64	13.5	64	49	1.3	4.16
21	Ogale	11.7	7.6	1.54	2.7	81	21	3.9	4.39
25	Ogale	9	8.2	1.1	N.D	28	7	4	3.33
26	Ogale	11.5	8	1.45	0.6	22	6	3.7	3.09
27	Ogale	12.5	8.5	1.47	0.004	62	19	3.3	4.13
33	Ebubu	12.1	9	1.35	B.D.L	44	9	4.9	3.78
34	Ebubu	11.8	9	1.33	B.D.L	10	6	1.7	2.3
35	Ebubu	13.5	6.9	1.97	0.7	148	42	3.5	5
39	Ebubu	13.6	10.6	1.28	B.D.L	28	8.4	3.3	3.33
49	Alode	10.3	9.2	1.13	2.9	55	12	4.6	4.01
EF5	Alesa	7.9	10.5	0.75	5.1	145	123	1.1	4.98
EF2	Alesa	- 0.5	1.9	-0.27	A.D.L	131	99	1.3	4.88

*ND* not determined, *BDL* below detection limit, *ADL* above detection limit

In municipal sewage samples (*n* = 2), δ^15^N and δ^18^O values varied largely, ranging from -0.5 to + 7.9‰ for δ^15^N, and + 1.9 to + 10.5‰ for δ^18^O.

## Discussion

### Source of NO_3_^−^ in the groundwater

The groundwater samples with elevated ions were predominantly from Alesa, Ogale, and Ebubu. The elevated ion (i.e., Na^+^, K^+^, Cl^−^, and Ca^2+^) levels in the groundwater and sewage reflect the anthropogenic influence related to the discharge of animal/human waste effluents in the area (Minet et al. 2017). Furthermore, several strong positive correlations existed between the NO_3_^−^ concentrations and the Na^+^, K^+^, Cl^−^, Sr^2+^, and Ca^2+^ concentrations. NO_3_^−^ had a strong positive correlation with Na^+^ (*R* = 0.9), indicating possible impacts from the municipal sewage on the NO_3_^−^ loading (Liu et al. 2006). Furthermore, NO_3_^−^ derived from septic effluents, human excreta, or animal wastes usually has a strong correlation with Cl^−^ (Liu et al. 2006). The groundwater NO_3_^−^ in this study generally shows a strong positive correlation with Cl^−^ (*R* = 0.94) across the study area. The correlation appears to be stronger in Alesa (*R* = 0.98), Ogale (*R* = 0.9), and Ebubu (*R* = 0.99) compared to Alode (*R* = 0.59) and Okochiri (*R* = 0.09). The Cl^−^ and NO_3_^−^ levels in the sewage were elevated, up to 242 mg/L and 58 mg/L, respectively. However, at the reference sites, Cl^−^ and NO_3_^−^ levels in the sewage were relatively low, 14 and 2.4 mg/L, respectively, suggesting that municipal sewage is likely the major contributing source of the NO_3_^−^ contamination in the groundwater. Meanwhile, the presence of pit latrine toilets containing leachates from human excreta may also contribute to the NO_3_^−^ levels in the groundwater. Also, NO_3_^−^ was positively correlated with K^+^ (*R* = 0.83), Sr^2+^ (*R* = 0.88) and Ca^2+^ (*R* = 0.87) concentrations. These correlations, however, were mostly weak (*R* values ranged from 0.05 to 0.2) in all the groundwater quality parameters at the reference site. Notably, NO_3_^−^ levels were consistently low (< 0.01 to 3 mg/L) at the reference sites. Therefore, the Na^+^, K^+^, Cl^−^, and Ca^2+^ concentrations suggest that the land use effect, i.e., leachate infiltration from domestic and municipal sewage and unlined pit latrine systems, is the point source of NO_3_^−^ loading (e.g., Minet et al. 2017).

Similarly, although the Mg^2+^, F^−^, and SO_4_^2−^ levels were relatively low in the groundwater and sewage, their concentrations exceeded the reference site values in several samples (Table 2S and 3S). NO_3_^−^ vs Mg had a strong positive correlation (*R* = 0.9), likewise NO_3_^−^ vs F^−^ (*R* = 0.53), and NO_3_^−^ vs SO_4_^2−^ (*R* = 0.64). Those elevated ion levels also indicated a possible anthropogenic influence, most likely sewage infiltration into the aquifer.

In line with the elevated ion concentration, Alesa, Ogale, and Ebubu groundwater showed an influence from anthropogenic activities (e.g., indiscriminate waste disposal into the municipal drainages). Leachates from municipal sewages often contain various contaminants, including salts and chloride compounds, which may infiltrate the aquifer (e.g., Aweto et al. 2023). The EC and TDS values were consistent with the findings by Eyankware et al. (2022) and Abam and Nwankwoala (2020). EC and TDS, which principally comprise Ca^2+^, Mg^2+^, K^+^, Na^+^, Cl^−^, SO_4_^2−^, HCO_3_^−^, and small amounts of dissolved organic matter (WHO 2017), strongly correlated with NO_3_^−^ (*R* = 0.93). Reference sites lacked such strong correlations, corroborating the influence of domestic and municipal sewage infiltration.

Furthermore, anthropogenic sources of NO_3_^−^ can be identified using the NO_3_^−^/Cl^−^ molar ratio since Cl^−^ is widely distributed in natural waters (Torres-Martínez et al. 2021). According to Anornu et al. (2017) and Liu et al. (2006), this approach compares the molar ratios for NO_3_^−^ and Cl^−^ with the assumption that halides, such as Cl^−^, are chemically inert when introduced into the environment. This property makes Cl^−^, which usually has minimal interaction with the subsoil (Guo et al. 2020), an ideal indicator of sewage, manure, and fertilizer when plotted against NO_3_^−^ (Gibrilla et al. 2020). Generally, groundwater with high values of Cl^−^ against low NO_3_^−^/Cl^−^ ratios are associated with NO_3_^−^ inputs from sewage and organic wastes, whereas high NO_3_^−^/Cl^−^ ratios with low Cl^−^ values suggest NO_3_^−^ inputs from agrochemicals (Anornu et al. 2017). Moreover, the NO_3_^−^/Cl^−^ ratio is low in groundwater unaffected by anthropogenic activities (Jiang et al. 2016). In this study, the NO_3_^−^/Cl^−^ ratios in the groundwater across Alesa, Ogale, and Ebubu overall were elevated, suggesting an anthropogenic influence. The relationship of the NO_3_^−^/Cl^−^ vs Cl^−^ concentration appears to be constant, implying that the groundwater has a consistent, non-variable source of NO_3_^−^ (Cao et al. 2021). All the samples showed significantly higher Cl^−^ levels with lower NO_3_^−^/Cl^−^ (Fig. 4a), suggesting that the NO_3_^−^ was derived from the ongoing anthropogenic activities in the area, which are likely leachates from the municipal sewage and the pit latrine systems. Interestingly, a subset of four samples deviated from the general trend, which we regard as an indication of nitrate removal in the study area, possibly due to denitrification (Fig. 4a).

Also, several scientific research has shown that dual NO_3_^−^ isotopes (i.e., δ^15^N-NO_3_^−^ and δ^18^O-NO_3_^−^) can assist in identifying NO_3_^−^ sources, as well as revealing the ongoing biogeochemical processes (e.g., nitrification and denitrification) in the groundwater (Biddau et al. 2023; Boumaiza et al. 2023; Degnan et al. 2016; He et al. 2022; Ju et al. 2023; Kendall et al. 2007; Mao et al. 2023). The δ^15^N-NO_3_^−^ and δ^18^O-NO_3_^−^ values for this study (Fig. 4b) are discussed below.

### Transport and fate of NO_3_^−^in the groundwater

#### Parallel occurrence of nitrification and denitrification?

While dual NO_3_^−^ isotopes have been widely used for NO_3_^−^ source assessment in various environments, a precise attribution is complicated by overlapping processes (e.g., nitrification and denitrification) (Granger & Wankel 2016), fractionation effects (Yu et al. 2020), and mixing of different sources (Harris et al. 2022). For each NO_3_^−^ source, there is a distinct dual isotope signature. For instance, δ^18^O and δ^15^N derived from nitrification of manure and sewage range from − 10 to + 15‰ and + 8 to + 25‰ for O and nitrogen isotopes, respectively (Kendall et al. 2007). In Alesa, Ogale, and Ebubu, groundwater DO content ranged from 1.5 to 8.9 mg/L, and such oxic conditions can favor nitrification as a nitrate source in the aquifer.

A first source attribution based on Kendall et al. (2007) shows that the data plotted in the “manure and sewage” zone (Fig. 4b). As mentioned, the communities with elevated NO_3_^−^ concentrations were characterized by drainage systems filled with domestic and municipal sewage (Fig. 7) and pit latrine toilets. Additional high NH_4_^+^ concentrations in the groundwater and sewage across the study communities can rapidly be converted to NO_3_^−^ by nitrifiers in oxic groundwater. The high NO_3_^−^/Cl^−^ molar ratios (> 1), as well as the elevated δ^15^N-NO_3_^−^ values (> 5) in the groundwater (Fig. 4c), further support that NH_4_^+^ from sewage or manure, is, upon nitrification, a significant source of NO_3_^−^ in the groundwater.

Thus, while nitrification appears to be the primary biogeochemical process across the three sites, there is evidence for simultaneous denitrification. Denitrification, regarded as all nitrate respiration processes, is vital for NO_3_^−^ removal in groundwater by transforming the dissolved NO_3_^−^ to N_2_O and N_2_ (Cantrell et al. 2007), as expressed in Eq. 1 below (Appelo & Postma 2005). It is, however, more likely to occur under limited oxygen conditions and available organic carbon (Xue et al. 2009).

1 $$N{O_{3^-}}_{(aq)}\rightarrow N{O_{2^-}}_{(aq)}\rightarrow NO\;(enzyme\;complex)\rightarrow N_2O_{\left(gas\right)}\rightarrow N_{2(gas)}$$

In this process, as the NO_3_^−^ decreases, both δ^15^N-NO_3_^−^ and δ^18^O-NO_3_^−^ of the NO_3_^−^ residual increase simultaneously due to the fractionation and enrichment of the ^18^O in the NO_3_^−^ (Harris et al. 2022; Jiang et al. 2016; Kendall 1998; Wassenaar 1994). The relationship between δ^15^N-NO_3_^−^ vs δ^18^O-NO_3_^−^, and δ^15^N-NO_3_^−^ or δ^18^O-NO_3_^−^ vs ln(NO_3_^−^) can provide information on the ongoing denitrification and mixing of NO_3_^−^ from different sources in the aquifer (Harris et al. 2022; Zakaria et al. 2023; Zaryab et al. 2023). Usually, groundwaters undergoing denitrification will exhibit a linear correlation between δ^15^N-NO_3_^−^ and δ^18^O- NO_3_^−^ (Jiang et al. 2016; Wassenaar 1994).

Despite the elevated groundwater DOC of up to 42 mg/L in Alesa, 49 mg/L in Ogale, 47 mg/L in Ebubu, and 54 mg/L in Alode, our results (i.e., lack of distinct positive or negative correlation between δ^15^N-NO_3_^−^ or δ^18^O-NO_3_^−^ and NO_3_^−^) suggest that denitrification is not the primary process for nitrogen transformation in the area (Zaryab et al. 2023). Given oxic conditions in most samples, this is plausible. In most samples, DO was above the threshold oxygen level for denitrification of 2 mg/L (Xue et al. 2012). Nevertheless, the build-up of NO_2_^−^ (0.2 to 2 mg/L, *n* = 8) and the observed slope of δ^15^N-NO_3_^−^ vs δ^18^O-NO_3_^−^ of 0.58 in the groundwater of Alesa and Ogale are indicators of potential denitrification. In groundwater, denitrification theoretically follows a dual isotope slope of 0.5 (Mayer et al. 2002).

**Fig. 5:**
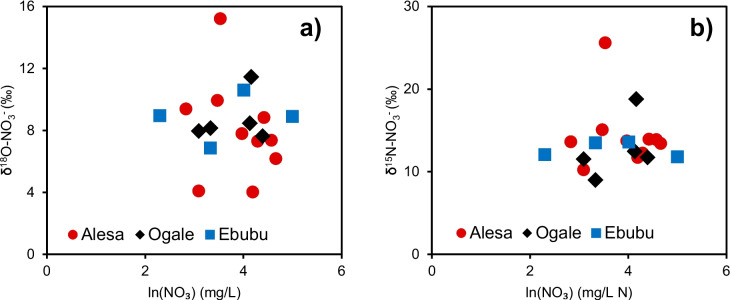
The plot of (**a**) δ^15^N-NO_3_^−^ vs ln(NO₃) (mg/L N), and (**b**) and δ^18^O-NO_3_^−^ vs ln(NO₃) (mg/L N) for Ogale and Ebubu in the study areas

Furthermore, in Alsea and Ogale, a strong positive correlation between δ^15^N-NO_3_^−^ and δ^18^O-NO_3_^−^ indicated the occurrence of biological fractionation, likely due to denitrification (Anornu et al. 2017). The weak negative correlations between δ^15^N-NO_3_^−^ and δ^18^O-NO_3_^−^ vs ln(NO_3_^−^) (Fig. 5a) suggested that the isotopic enrichment of NO_3_^−^ in the groundwater should have been caused by denitrification rather than dilution or mixing of NO_3_^−^ from different sources (Xia et al. 2017). Also, the ratio of δ^15^N-NO_3_^−^ vs δ^18^O-NO_3_^−^ varied between 1.45 to 2.92. Those values are consistent with the reported ratios for groundwater denitrification, suggesting that simultaneous re-oxidation of NO_2_^−^ occurred concurrently with NO_3_^−^ reduction (Harris et al. 2022).

**Fig. 6:**
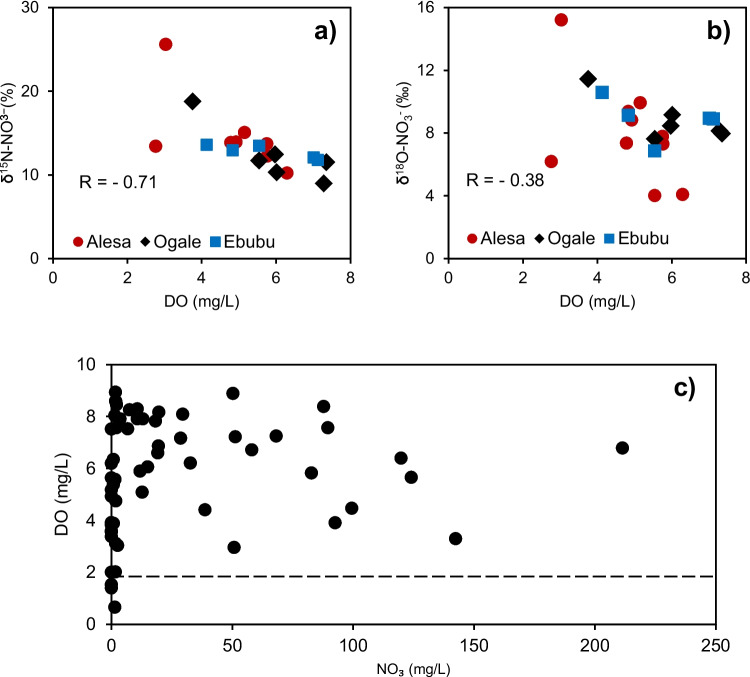
The plot of (**a**) δ^15^N-NO_3_^−^ vs DO, (**b**) δ^18^O-NO_3_^−^ vs DO, and (**c**) DO vs NO_3_^−^ in the study area

Also, DO appears to play a vital role in controlling denitrification in Alesa, Ogale, and Ebubu. Based on the thermodynamic principle, a complete depletion of oxygen is required for denitrification to proceed. Clearly, the NO_3_^−^ decreased sharply when DO is ≤ 2 mg/L (Fig. 6c). Also, δ^15^N-NO_3_^−^ composition in the groundwater increases with decreasing DO (Fig. 6a, R =  − 0.71), suggesting that DO is an overarching control on denitrification in the groundwater. A similar but weak negative correlation (Fig. 6b, R =  − 0.38) is evident in the δ^18^O-NO_3_^−^ vs DO plot, across the three communities.

In contrast, there was no relationship between DOC concentration and δ^15^N-NO_3_^−^ in the groundwater or between DOC concentration and NO_3_^−^. This suggests that the dissolved C fraction was not consumed during denitrification. The lack of a clear correlation between DOC and δ^15^N-NO_3_^−^ is consistent with the findings of Hinkle et al. (2007). They suggested that the dissolved carbon fraction is less relevant for denitrification than solid-phase organic carbon within the aquifer matrix. All the groundwater samples had elevated DOC levels of up to 54 mg/L, which we attribute to the ongoing oil and gas extraction activities in the Niger Delta. While such high DOC may not be consumed directly during denitrification, the labile organic carbon can act as a potential electron donor during groundwater denitrification by providing the necessary electrons needed for the reduction of NO_3_^−^ or NO_2_^−^ to N_2_O or N_2_ under depleted oxygen condition. However, no conclusive evidence showed that DOC, as an electron donor, controlled the denitrification process in the study area.

**Fig. 7:**
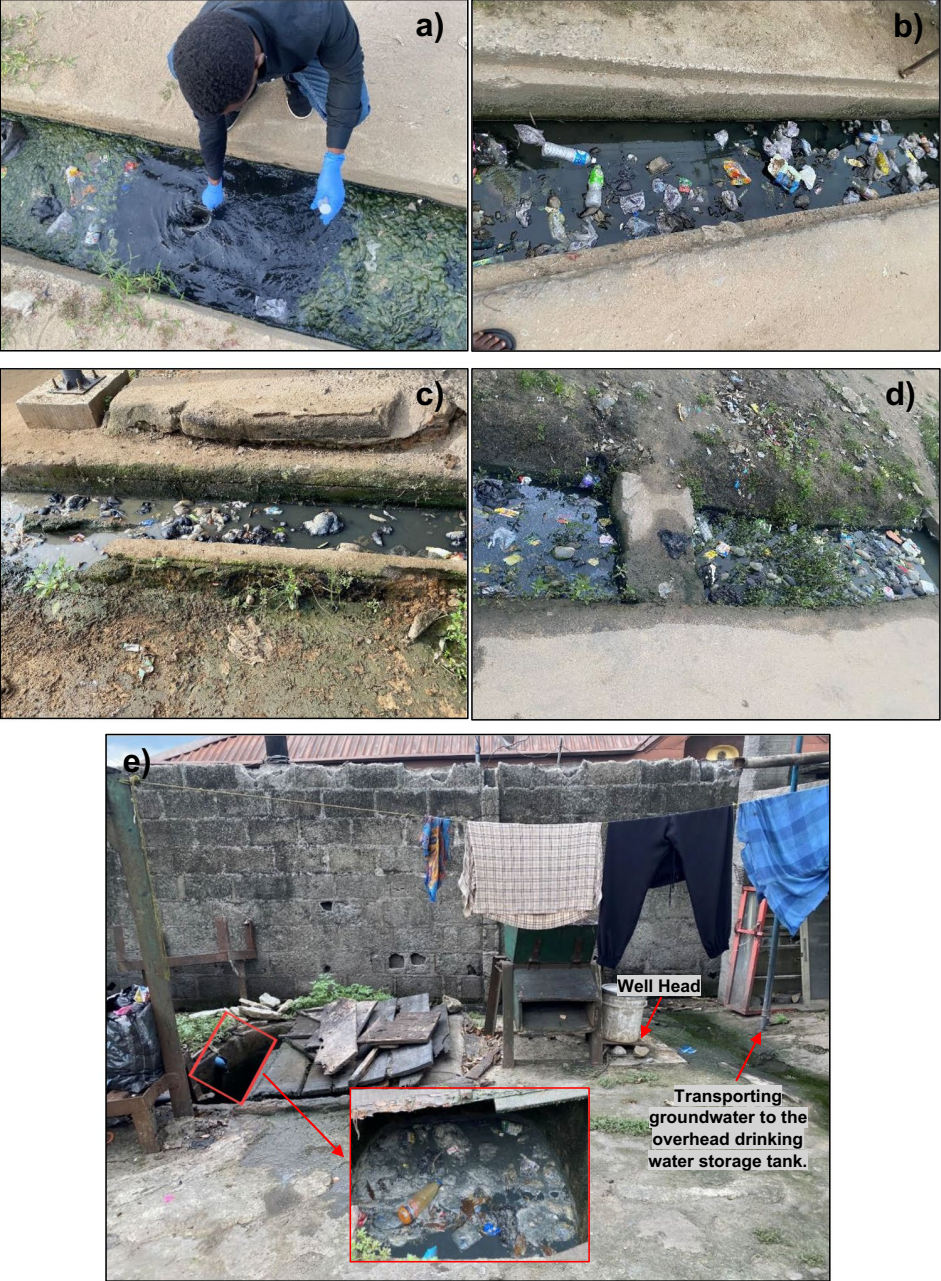
**a, b** Municipal sewage in Alesa, (**c, d**) municipal sewage in Ogale with restricted flow, and (**e**) domestic sewage in Alesa with restricted flow. During rainfall, the sewage is transported to various parts of the community, infiltrating the aquifer. In flooding events, the sewage is transported to the nearby surface waters

Furthermore, despite the elevated DOC concentrations due to the heavy impacts of the oil and gas activities in the area, the DO level is still high. This is either due to (1) continuous recharge from precipitation or (2) the absence of aerobic respiration or that DOC is not bioavailable for microbial respiration. With a 2800 to 4000-mm/year precipitation rate (Ohwoghere-Asuma et al. 2023), DO, through the soil, is continuously introduced into the shallow and sandy aquifer of the investigated sites. Given the permeable and sandy nature of the soils and the low water Table (1 to 11 m) in the areas, oxygen consumed in the soil zone is resupplied by gaseous oxygen transport through the soil, resulting in insignificant oxygen consumption (e.g., Appelo & Postma 2005). Furthermore, according to Rajendiran et al. (2023), in oxic groundwater where aerobic respiration is present, DO shows an inverse correlation with DOC. This relationship, however, depends on the bioavailability of the DOC (Chapelle et al. 2012). Nevertheless, in this study, DO poorly correlates with DOC, indicating either the absence of aerobic respiration or that DOC is not bioavailable for microbial respiration. Notably, the high groundwater temperature (up to 32.5 °C), which affects the groundwater saturation level, appears to limit the DOC degradation potential in the investigated areas (Jindrová et al. 2002). Usually, DOC degradation depletes DO. When DO is completely used up, electron acceptors such as NO_3_^−^, Mn^4+^, Fe^3+^, and SO_4_^2−^, if available, will further oxidize DOC (Christensen et al. 2000). The low concentrations of Fe and Mn in this study are due to the dominance of quartz grains (95 to 99%) in the aquifer (Nwajide 2013) and less anthropogenic activities capable of releasing Fe and Mn into the aquifer. Nevertheless, the impact of the oil and gas industry released Fe in a portion of the Ogale, causing the occurrence of suboxic conditions (i.e., DO < 2 mg/L, NO_3_^−^  < 0.5 mg/L and Fe ≥ 0.1 mg/L (Tesoriero et al. 2024)) in the affected portion. Here, the rusting of an underground NNPCL petroleum pipeline was observed as shown by the accumulation of Fe precipitates (reddish-brown rust particles) as stains on surfaces of (1) polyvinyl chloride overhead tanks used for storage of drinking water and (2) plumbing fixtures, as well as other domestic water containers in residential homes next to the underground pipeline (Fig. S5). As a result, Fe concentrations were elevated, up to 50 mg/L in 2022 and 46 mg/L in 2023, while DO levels were low (Table 2S), prompting reducing conditions for NO_3_^−^ and therefore denitrification observed in few samples in the Ogale, hence the low nitrate concentrations in those samples (Fig. 1) and Fig. 7.

#### Biogeochemical processes of redox reaction on nitrogen (N) behavior in the groundwater

**Fig. 8:**
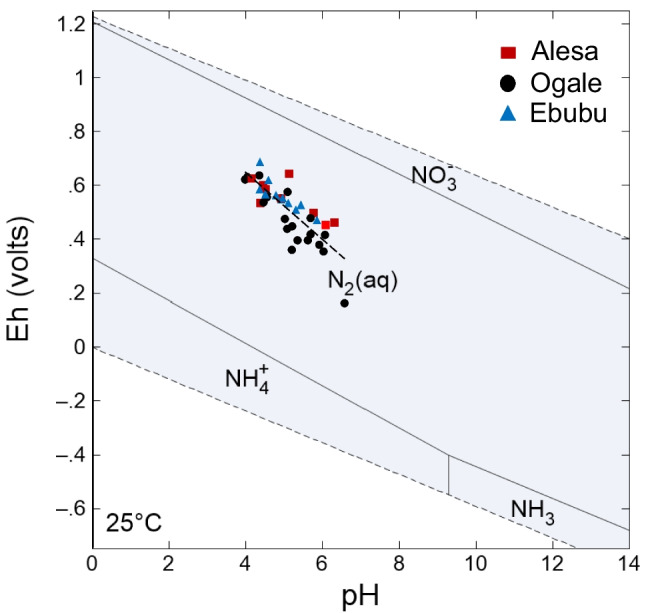
Eh–pH for nitrogen species in Alesa, Ogale, and Ebubu groundwater. The diagram was generated using the Geochemist’s Workbench software (17.0 edition)

The oxidation/reduction (redox) reaction potential (Eh) is fundamental for most geochemical processes in aqueous environments. While nitrogen compounds actively undergo biogeochemical reactions in groundwater, changes in Eh and pH conditions control the occurrence and stability of the various nitrogen species (i.e., NO_3_^−^, NO_2_^−^, and NH_4_^+^) (Lidman et al. 2017; Reddy & D'angelo 1997). In the Eh–pH diagram (Fig. 8), aqueous species of nitrogen in groundwater under standard conditions (25 °C and 1 atm) are dominated by NO_3_^−^ under highly oxidizing conditions, NH_4_^+^ under highly reducing conditions, and NH_3_^+^ under highly basic and reducing conditions. At the same time, N_2_ occupies a large area due to atmospheric influence (Fig. 8). In this study, changes in Eh and pH have been identified as an important controlling factor for the dominance of a particular aqueous species of nitrogen predicted to be present at 25 °C with an activity value of 1 × 10^−3^ M dissolved nitrogen in the groundwater. The 1 × 10^−3^ M used is the NO_3_^−^ activity value commonly found in NO_3_^−^ polluted groundwaters (Appelo & Postma 2005). It is, however, essential to note that changes in pressure do not necessarily introduce substantial errors in the Eh–pH boundaries calculated for 1 bar. Similarly, the influence of temperature on nitrogen transformation is usually in the same direction (e.g., the rate of chemical reaction speeds up with high temperature) (e.g., Thiagalingam & Kanehiro 1973). This implies that slight fluctuations in temperature from 25 to 29 °C may not significantly alter the stability fields in the Eh–pH diagram (Fig. 8).

The Eh values in the Alesa, Ogale, and Ebubu groundwater ranged from 113 to 641 mV (pH = 4 to 6.6), where the NO_3_^−^ contamination was observed, and higher in Alode and Okochiri (Eh = 117 to 801 mV, pH = 4.4 to 6.9), where NO_3_^−^ levels were relatively low. Both sites are characterized by highly oxidizing conditions favorable for nitrification. This explains the high NO_3_^−^ concentrations in the groundwater (Takatert et al. 1999; Zhao et al. 2016). As shown in Fig. 8, the groundwater samples plotted in the field of N_2(aq)_ stability between the boundary lines for NH_4_^+^ and NO_3_^−^. This supports the idea that nitrifying and denitrifying could be possible in groundwater at our study sites. This result is consistent with a similar investigation in the coastal aquifer of Lagos, Nigeria (Aladejana et al. 2020).

### NO_3_^−^ export potential and management implications

**Fig. 9:**
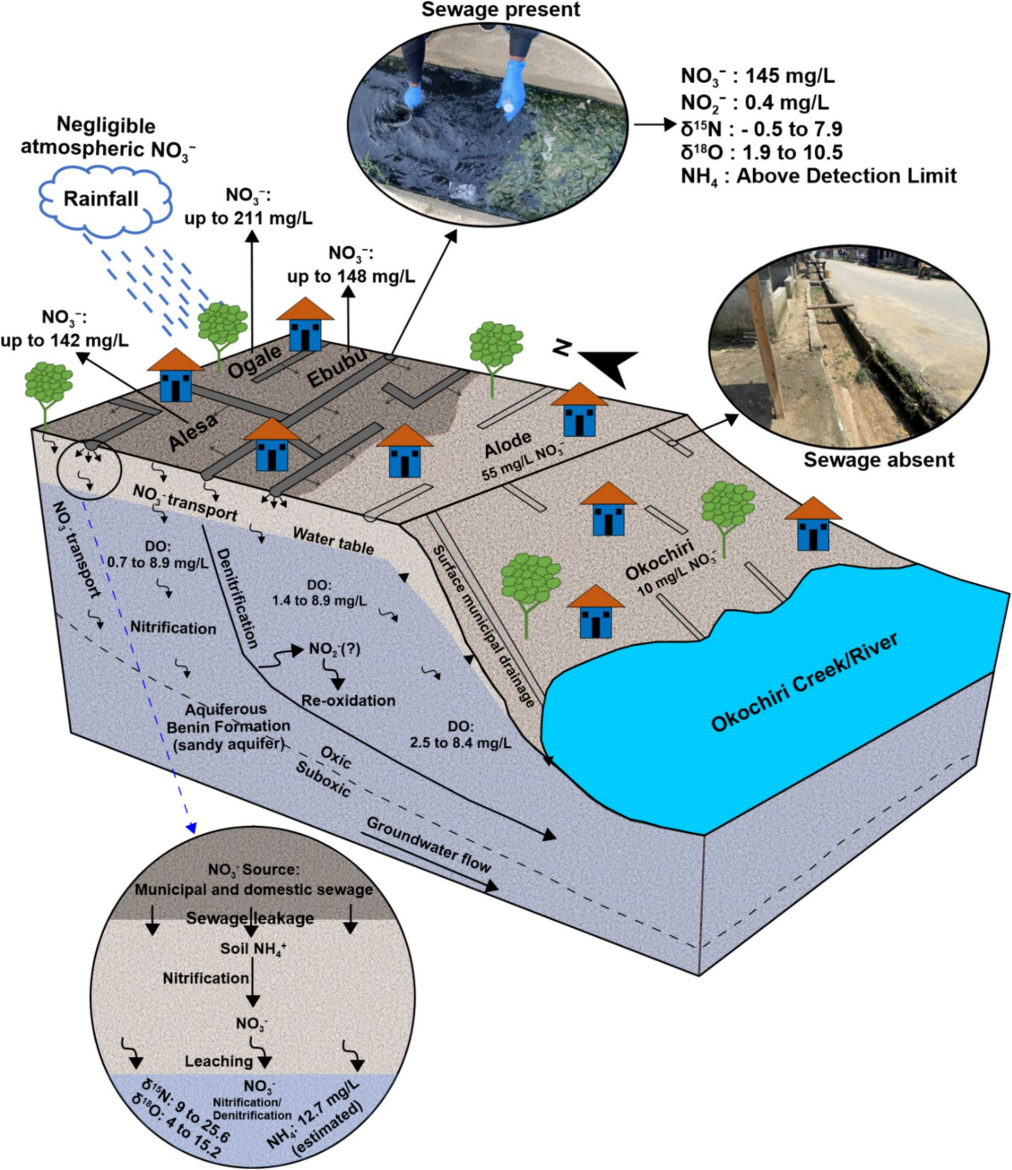
Schematic diagram (not to scale) of the NO_3_^−^ source, transport, and fate in the groundwater of the eastern Niger Delta Region of Nigeria

The schematic diagram of the NO_3_^−^ source in the groundwater of the eastern Niger Delta is given in Fig. 9. The sewage availability, DO, and groundwater flow direction were the controlling factors influencing the distribution of NO_3_^−^. The nitrogen derived from the sewage likely migrated into the aquifer where it was subsequently nitrified. The NO_3_^−^ was transported along the groundwater flow direction within the groundwater system. Groundwater level in the oxic aquifers upgradient is relatively high compared to the oxic aquifers downgradient (1.5 m). Such differences have created pathways for NO_3_^−^ to be transported, thereby presenting the potential for export to the nearby Okochiri River.

During the 2022 and 2023 sampling campaigns, some minor flooding occurred, due to the frequent heavy rainfall, improper design and maintenance of the drainage channels, and blockage of the municipal drainages. Consequently, sewage from the various drainages was transported to other areas within the community when the soil infiltration capacity was exceeded. This could cause a continuous rise in the groundwater levels facilitating the export of NO_3_^−^ from shallow groundwater to the Okochiri River. Although the groundwater has shown evidence of denitrification, the prevailing redox conditions and the high groundwater NO_3_^−^ load did not support a complete attenuation of NO_3_^−^ in the affected communities. Denitrification, therefore, should not be relied upon for the effective NO_3_^−^ reduction. Also, anthropogenic activities responsible for the elevated groundwater NO_3_^−^ are still ongoing, posing the risk of further increases in the groundwater NO_3_^−^ concentration. Hence, there is a need for urgent groundwater management measures to protect the groundwater.

The management measures should focus on the following:

1. Safe domestic and municipal sewage management practices should be introduced to reduce the amount of anthropogenic nitrogen reaching the aquifer via municipal and domestic sewage infiltration into the groundwater. Furthermore, responsible municipal and domestic sewage disposal will assist in freeing the clogged drainages, reducing the frequently occurring flooding and minimizing the potential spread of NO_3_^−^ contamination.
2. Measures to encourage immediate discontinuous use of the contaminated PSW while alternative safe drinking water sources (e.g., sachet or bottled) are explored. The groundwater NO_3_^−^ levels should, however, be monitored continuously to ensure that NO_3_^−^ levels are within safe limits.

## Limitations and needs for further study

Since exposure to high levels of NO_3_^−^ is dangerous to human health, the role of drinking water NO_3_^−^ exposure in the Alesa, Ogale, Ebubu, and Alode as a risk factor for specific cancers, adverse reproductive outcomes, and other adverse health effects should be investigated. Particularly, since NO_3_^−^ is not the only probelmatic contaminant in the area (Aleku et al. 2024). The findings from such investigations will provide public policymakers with a comprehensive understanding of the true health burden associated with NO_3_^−^ contamination in the eastern Niger Delta region. Also, since the prevailing redox conditions do not support effective denitrification in the affected communities, further studies on various NO_3_^−^ removal techniques, including chemical and biological denitrification, ion exchange, reverse osmosis, and adsorption, that use greener nanotechnologies (e.g., nanocomposites and nanorods) should be investigated and used for complete NO_3_^−^ removal in the area.

## Conclusion

The study revealed that the groundwater of Alesa, Ogale, and Ebubu in the eastern Niger Delta is contaminated with NO_3_^−^, at levels up to 142 mg/L, 211 mg/L, and 148 mg/L, respectively. The groundwater NO_3_^−^ concentration decreased downgradient in Alode (55 mg/L) and Okochiri (10 mg/L). Similarly, NO_2_^−^ groundwater contamination was observed in a few samples at up to 2 mg/L. To further assess the source of NO_3_^−^, we applied a dual isotope (δ^15^N-NO_3_^−^ and δ^18^O-NO_3_^−^) and hydrochemical markers (major ions and NO_3_^−^/Cl^−^ ratio) approach. Our isotopic data were consistent with a sewage source of groundwater NO_3_^−^. It also showed that nitrification is the primary biogeochemical process controlling the groundwater NO_3_^−^ levels. Our hydrochemical markers also revealed that the NO_3_^−^ contamination is derived from sewage effluents, which likely released N-containing compounds before being nitrified. While nitrification is the primary ongoing biogeochemical process, the data also revealed that denitrification co-occurs in the groundwater, especially in Alesa and Ogale. Given the oxidizing condition of the groundwater, denitrification should not be relied upon for the complete attenuation of NO_3_^−^ in the affected communities. Therefore, there is an urgent need to introduce safe domestic and municipal sewage management practices to protect groundwater. This will also prevent the potential NO_3_^−^ movement into the surface and nearshore seawater.

## Supplementary Information

Below is the link to the electronic supplementary material.

Supplementary file1 (DOCX 12827 KB)

## Data Availability

All data generated or analyzed during this study are included in this published article (and its supplementary information files).
