# Supplementary material for: Source, transport, and fate of nitrate in shallow groundwater in the eastern Niger Delta
Source: Environ Sci Pollut Res Int. 2024 Nov 20;31(56):65034–50. doi: 10.1007/s11356-024-35499-6 (PMC11624242; doi:10.1007/s11356-024-35499-6)
Supplement: Supplementary file 1 — Supplementary file1 (DOCX 12827 KB) [file 11356_2024_35499_MOESM1_ESM.docx]

**Supplementary Data**

**Table 1S: Physico-chemical parameters of groundwater in the study area (2022 and 2023)**

| **ID** | **Site** | **Longitude** | **Latitude** | **Altitude (m)** | **pH** | **Eh (mV)** | **DO (mg/L)** | **DO (%)** | **EC (µS/cm)** | **TDS (mg/L)** | **Salinity (PSU)** | **Temp. (°C)** | **Alkalinity (mg/L)** |
| --- | --- | --- | --- | --- | --- | --- | --- | --- | --- | --- | --- | --- | --- |
| **1–22** | Alesa | 7.112087 | 4.796872 | 20.6 | 5.8 | 451 | 3.3 | 43 | 512 | 256 | 0.2 | 27.2 | 3 |
| **1–23** | Alesa | 7.112087 | 4.796872 | 20.6 | 5.8 | ND | 2.8 | 37 | 395 | 198 | 0.1 | 30.5 | 31 |
| **2–22** | Alesa | 7.120062 | 4.788458 | 24.7 | 5.1 | 596 | 4.4 | 57 | 228 | 114 | 0.1 | 28.3 | 0 |
| **2–23** | Alesa | 7.120062 | 4.788458 | 24.7 | 4.7 | ND | 3 | 39 | 187 | 93 | 0.1 | 29.3 | 1 |
| **3–22** | Alesa | 7.120909 | 4.795887 | 37.5 | 4.5 | 538 | 5.8 | 77 | 302 | 151 | 0.1 | 29.1 | 3 |
| **3–23** | Alesa | 7.120909 | 4.795887 | 37.5 | 4.6 | ND | 4.9 | 64 | 215 | 108 | 0.1 | 29.3 | 0 |
| **4–22** | Alesa | 7.104822 | 4.794335 | 12.1 | 6.3 | 414 | 6.6 | 83 | 185 | 91 | 0.1 | 26.5 | 4 |
| **4–23** | Alesa | 7.104822 | 4.794335 | 12.1 | 4.7 | ND | 4.8 | 64 | 77 | 38 | 0.1 | 30.1 | 0 |
| **5–22** | Alesa | 7.106808 | 4.799022 | 21.1 | 4.4 | 488 | 7.6 | 97 | 327 | 163 | 0.2 | 27.5 | 0 |
| **5–23** | Alesa | 7.106808 | 4.799022 | 21.1 | 4.5 | ND | 5.8 | 77 | 245 | 122 | 0.2 | 30.5 | 0 |
| **6–22** | Alesa | 7.111695 | 4.78944 | 5.1 | 4.5 | 490 | 6.7 | 86 | 321 | 160 | 0.2 | 28.1 | 0 |
| **6–23** | Alesa | 7.111695 | 4.78944 | 5.1 | 4.4 | ND | 5.8 | 76 | 192 | 96 | 0.1 | 30.4 | 0 |
| **7–22** | Alesa | 7.109185 | 4.800108 | 23.6 | 4.2 | 579 | 4.5 | 57 | 400 | 200 | 0.2 | 27.6 | 0 |
| **7–23** | Alesa | 7.109185 | 4.800108 | 23.6 | 4.2 | ND | 4.8 | 63 | 322 | 161 | 0.2 | 28.8 | 0 |
| **8–22** | Alesa | 7.118945 | 4.78445 | 25.1 | 4.9 | 501 | 6.2 | 78 | 165 | 82 | 0.1 | 26.6 | 1 |
| **8–23** | Alesa | 7.118945 | 4.78445 | 25.1 | 4.7 | ND | 5.2 | 67 | 125 | 63 | 0.1 | 29.4 | 3 |
| **9–22** | Alesa | 7.120667 | 4.787682 | 16.5 | 6.1 | 405 | 7.3 | 93 | 298 | 147 | 0.1 | 28.3 | 14 |
| **9–23** | Alesa | 7.120667 | 4.787682 | 16.5 | 5.1 | ND | 5.5 | 73 | 244 | 122 | 0.1 | 29.9 | 14 |
| **10–22** | Alesa | 7.122352 | 4.78513 | 24.8 | 4.5 | 554 | 7.8 | 100 | 90 | 45 | 0.04 | 27.7 | 0 |
| **10–23** | Alesa | 7.122352 | 4.78513 | 24.8 | 3.5 | ND | 6.3 | 85 | 82 | 41 | 0.04 | 31.1 | 0 |
| **Minimum** | | | | | 3.5 | 405 | 2.8 | 37 | 77 | 38 | 0.04 | 26.5 | 0 |
| **Maximum** | | | | | 6.3 | 596 | 7.8 | 100 | 512 | 256 | 0.2 | 31.1 | 31 |
| **Median** | | | | | 4.7 | 496 | 5.7 | 75 | 236 | 118 | 0.1 | 29 | 0 |
| **Average** | | | | | 4.8 | 502 | 5.5 | 71 | 246 | 123 | 0.1 | 28.8 | 3.7 |
| **11–22** | Ogale | 7.128107 | 4.788077 | 14.4 | 5.4 | 348 | 2 | 26 | 67 | 33 | 0.03 | 27.8 | 15 |
| **12–22** | Ogale | 7.129307 | 4.795973 | 15.8 | 6.6 | 113 | 2.3 | 29 | 364 | 183 | 0.2 | 28.1 | 94 |
| **13–22** | Ogale | 7.128767 | 4.78765 | 26.3 | 6 | 307 | 3 | 39 | 162 | 81 | 0.1 | 27.7 | 17 |
| **13–23** | Ogale | 7.128767 | 4.78765 | 26.3 | 6.1 | ND | 0.7 | 9 | 206 | 103 | 0.1 | 30.7 | 100 |
| **14–22** | Ogale | 7.128502 | 4.787285 | 8 | 5.9 | 331 | 3.9 | 50 | 520 | 260 | 0.3 | 27.5 | 45 |
| **15–22** | Ogale | 7.120435 | 4.787102 | 17.1 | 5.6 | 348 | 5.6 | 71 | 126 | 62 | 0.1 | 27.1 | 2.5 |
| **15–23** | Ogale | 7.120435 | 4.787102 | 17.1 | 5.5 | ND | 3.8 | 48 | 374 | 187 | 0.2 | 29.2 | 35 |
| **16–22** | Ogale | 7.127315 | 4.786512 | 19.3 | 5.1 | 391 | 1.53 | 19 | 67 | 33 | 0.03 | 26.5 | 2 |
| **16–23** | Ogale | 7.127315 | 4.786512 | 19.3 | 5.6 | ND | 5.1 | 69 | 31 | 16 | 0.01 | 30.1 | 2 |
| **17–22** | Ogale | 7.126735 | 4.787023 | 15.7 | 6.1 | 368 | 6.8 | 87 | 852 | 427 | 0.4 | 27.6 | 0 |
| **17–23** | Ogale | 7.126735 | 4.787023 | 15.7 | 4.4 | ND | 4.7 | 60 | 17 | 8 | 0.1 | 28.8 | 1 |
| **18–22** | Ogale | 7.12799 | 4.789133 | 11.9 | 5.7 | 431 | 5.9 | 75 | 100 | 50 | 0.1 | 27.3 | 24 |
| **19–22** | Ogale | 7.132052 | 4.791505 | 12.4 | 5 | 428 | 3.1 | 38 | 35 | 18 | 0.02 | 25.1 | 0 |
| **19–23** | Ogale | 7.132052 | 4.791505 | 12.4 | 4.1 | ND | 3.4 | 45 | 41 | 20 | 0.02 | 30 | 0 |
| **20–22** | Ogale | 7.126753 | 4.781363 | 14.1 | 4 | 584 | 5.7 | 73 | 371 | 185 | 0.2 | 28.6 | 0 |
| **20–23** | Ogale | 7.126753 | 4.781363 | 14.1 | 4.7 | ND | 4.4 | 58 | 20 | 10 | 0.01 | 29.2 | 0 |
| **21–22** | Ogale | 7.133773 | 4.778295 | 22.2 | 5.1 | 529 | 8.2 | 103 | 91 | 46 | 0.04 | 27.7 | 0 |
| **21–23** | Ogale | 7.133773 | 4.778295 | 22.2 | 3.9 | ND | 5.5 | 73 | 230 | 115 | 0.1 | 29.5 | 0 |
| **22–22** | Ogale | 7.12643 | 4.788903 | 26.2 | 5.2 | 313 | 3.6 | 45 | 55 | 27 | 0.02 | 27.6 | 0 |
| **23–22** | Ogale | 7.126427 | 4.789248 | 20.8 | 5.2 | 401 | 3.9 | 74 | 60 | 30 | 0.03 | 26.7 | 0 |
| **23–23** | Ogale | 7.126427 | 4.789248 | 20.8 | 5.2 | ND | 4.2 | 55 | 32 | 16 | 0.01 | 29.2 | 0 |
| **24–22** | Ogale | 7.133198 | 4.775068 | 30.1 | 4.6 | 509 | 7.2 | 93 | 105 | 53 | 0.1 | 28.7 | 0 |
| **24–23** | Ogale | 7.133198 | 4.775068 | 30.1 | 5 | ND | 3.8 | 51 | 32 | 16 | 0.01 | 29 | 0 |
| **25–22** | Ogale | 7.12786 | 4.778627 | 19.9 | 4.4 | 591 | 8.1 | 106 | 118 | 59 | 0.1 | 28.9 | 0 |
| **25–23** | Ogale | 7.12786 | 4.778627 | 19.9 | 4 | ND | 7.3 | 91 | 87 | 44 | 0.04 | 27.1 | 0 |
| **26–22** | Ogale | 7.126795 | 4.783217 | 16.3 | 4 | 575 | 8.4 | 107 | 294 | 147 | 0.1 | 27.7 | 0 |
| **26–23** | Ogale | 7.126795 | 4.783217 | 16.3 | 4 | ND | 7.4 | 93 | 78 | 39 | 0.04 | 27.9 | 0 |
| **27–22** | Ogale | 4.787298 | 7.128548 | 11.5 | 5.7 | 371 | 3 | 36 | 327 | 163 | 0.2 | 27.5 | 17 |
| **27–23** | Ogale | 4.787298 | 7.128548 | 11.5 | 4.1 | ND | 6 | 80 | 172 | 86 | 0.1 | 30.1 | 0 |
| **Minimum** | | | | | 3.9 | 113 | 0.7 | 9 | 17 | 8 | 0.01 | 25.1 | 0 |
| **Maximum** | | | | | 6.6 | 591 | 8.4 | 107 | 852 | 427 | 0.4 | 30.7 | 100 |
| **Median** | | | | | 5.1 | 391 | 4.4 | 60 | 100 | 50 | 0.1 | 27.9 | 0 |
| **Average** | | | | | 5 | 408 | 4.7 | 62 | 174 | 87 | 0.1 | 28 | 12 |
| **28–22** | Ebubu | 7.155532 | 4.75196 | 7.3 | 5.1 | 487 | 8.6 | 109 | 21 | 10 | 0.01 | 27.9 | 2 |
| **29–22** | Ebubu | 7.156438 | 4.758375 | 17.2 | 4.4 | 538 | 3.4 | 43 | 341 | 171 | 0.2 | 27.8 | 0 |
| **30–22** | Ebubu | 7.150593 | 4.781777 | 15.9 | 4.6 | 573 | 7.9 | 103 | 81 | 41 | 0.04 | 28.5 | 0 |
| **31–22** | Ebubu | 7.159867 | 4.783162 | 21.9 | 4.9 | 505 | 8.9 | 115 | 38 | 18 | 0.02 | 27.9 | 0 |
| **31–23** | Ebubu | 7.159867 | 4.783162 | 21.9 | 3.5 | ND | 7.5 | 97 | 51 | 25 | 0.02 | 29.5 | 0 |
| **32–22** | Ebubu | 7.157197 | 4.77655 | 4.4 | 4.5 | 520 | 7.2 | 94 | 154 | 76 | 0.1 | 28.8 | 0 |
| **33–22** | Ebubu | 7.151137 | 4.77939 | 14.5 | 4.8 | 516 | 8.3 | 105 | 73 | 36 | 0.03 | 27.5 | 0 |
| **33–23** | Ebubu | 7.151137 | 4.77939 | 14.5 | 4.6 | ND | 7 | 92 | 119 | 60 | 0.1 | 28.9 | 0 |
| **34–22** | Ebubu | 7.150657 | 4.776055 | 14.8 | 5.4 | 480 | 6.4 | 84 | 447 | 225 | 0.2 | 29.1 | 4 |
| **34–23** | Ebubu | 7.150657 | 4.776055 | 14.8 | 5.2 | ND | 7.5 | 97 | 376 | 188 | 0.2 | 29.5 | 3 |
| **35–22** | Ebubu | 7.146193 | 4.77285 | 16.3 | 5.9 | 424 | 6.1 | 78 | 93 | 46 | 0.04 | 28.5 | 8 |
| **35–23** | Ebubu | 7.146193 | 4.77285 | 16.3 | 5.2 | ND | 5.5 | 72 | 376 | 188 | 0.2 | 28.3 | 3 |
| **36–22** | Ebubu | 7.153428 | 4.771623 | 11.6 | 5.3 | 462 | 8.3 | 106 | 72 | 35 | 0.03 | 27.7 | 1 |
| **ID** | **Site** | **Longitude** | **Latitude** | **Altitude (m)** | **pH** | **Eh (mV)** | **DO (mg/L)** | **DO (%)** | **EC (µS/cm)** | **TDS (mg/L)** | **Salinity (PSU)** | **Temp. (°C)** | **Alkalinity (mg/L)** |
| **36–23** | Ebubu | 7.153428 | 4.771623 | 11.6 | 3.6 | ND | 7.1 | 94 | 59 | 29 | 0.03 | 29.2 | 0 |
| **37–22** | Ebubu | 7.147802 | 4.766063 | 27.3 | 5 | 506 | 8.6 | 112 | 41 | 20 | 0.02 | 29 | 0 |
| **38–22** | Ebubu | 7.138585 | 4.783827 | 16.6 | 4.4 | 641 | 6.9 | 87 | 84 | 42 | 0.04 | 27.2 | 0 |
| **38–23** | Ebubu | 7.138585 | 4.783827 | 16.6 | 4.1 | ND | 4.1 | 53 | 86 | 43 | 0.04 | 28.8 | 0 |
| **39–22** | Ebubu | 7.150598 | 4.781781 | 16.6 | 4.9 | 506 | 7.9 | 103 | 81 | 41 | 0.04 | 27.9 | 0 |
| **39–23** | Ebubu | 7.150598 | 4.781781 | 16.6 | 4.1 | ND | 6.4 | 83 | 72 | 31 | 0.02 | 29 | 0 |
| **Minimum** | | | | | 3.5 | 424 | 3.4 | 43 | 21 | 10 | 0.01 | 27.2 | 0 |
| **Maximum** | | | | | 5.9 | 641 | 8.9 | 115 | 447 | 225 | 0.2 | 29.5 | 8 |
| **Median** | | | | | 4.8 | 506 | 7.2 | 94 | 81 | 41 | 0.04 | 28.5 | 0 |
| **Average** | | | | | 4.7 | 513 | 7 | 91 | 140 | 70 | 0.07 | 28.5 | 1.1 |
| **40–22** | Alode | 7.1099 | 4.765987 | 16.6 | 4.9 | 561 | 7.5 | 95 | 20 | 10 | 0.01 | 27.1 | 0 |
| **40–23** | Alode | 7.1099 | 4.765987 | 16.6 | 6.1 | ND | 1.4 | 19 | 64 | 32 | 0.03 | 30.5 | 22 |
| **41–22** | Alode | 7.115575 | 4.760697 | 18.8 | 5 | 426 | 6.4 | 81 | 100 | 50 | 0.1 | 27.2 | 1 |
| **42–22** | Alode | 7.122632 | 4.776868 | 23.7 | 5.3 | 118 | 3.6 | 49 | 63 | 31 | 0.03 | 30.6 | 4 |
| **42–23** | Alode | 7.122632 | 4.776868 | 23.7 | 5.3 | ND | 6.8 | 94 | 47 | 24 | 0.02 | 31 | 5 |
| **43–22** | Alode | 7.123132 | 4.776448 | 14.4 | 6.3 | 214 | 2 | 26 | 351 | 175 | 0.2 | 28 | 62 |
| **44–22** | Alode | 7.12319 | 4.778118 | 23.4 | 4.9 | 669 | 5.1 | 67 | 87 | 44 | 0.04 | 28.9 | 6 |
| **44–23** | Alode | 7.12319 | 4.778118 | 23.4 | 5 | ND | 7.9 | 100 | 53 | 27 | 0.03 | 29.1 | 1 |
| **45–22** | Alode | 7.121092 | 4.774338 | 25.3 | 4.8 | 374 | 5.6 | 74 | 71 | 36 | 0.03 | 28.6 | 0 |
| **45–23** | Alode | 7.121092 | 4.774338 | 25.3 | 5.4 | ND | 5.5 | 76 | 35 | 17 | 0.02 | 30.7 | 3 |
| **46–22** | Alode | 7.120805 | 4.774332 | 6.5 | 5.1 | 418 | 4.9 | 64 | 38 | 19 | 0.02 | 28.4 | 1 |
| **46–23** | Alode | 7.120805 | 4.774332 | 6.5 | 5 | ND | 6.2 | 85 | 30 | 15 | 0.01 | 30.5 | 0 |
| **47–22** | Alode | 7.12017 | 4.772995 | 21.7 | 4.8 | 435 | 6.2 | 83 | 56 | 28 | 0.02 | 29.9 | 0 |
| **47–23** | Alode | 7.12017 | 4.772995 | 21.7 | 5.2 | ND | 5.9 | 81 | 20 | 10 | 0.01 | 30.9 | 1 |
| **48–22** | Alode | 7.118968 | 4.77318 | 27.5 | 5.1 | 293 | 3.8 | 50 | 36 | 18 | 0.02 | 29 | 1 |
| **48–23** | Alode | 7.118968 | 4.77318 | 27.5 | 5.1 | ND | 5.6 | 77 | 20 | 10 | 0.01 | 30.8 | 1 |
| **49–22** | Alode | 7.118422 | 4.774342 | 17.2 | 4.8 | 473 | 5.2 | 74 | 59 | 30 | 0.03 | 33.5 | 0 |
| **49–23** | Alode | 7.118422 | 4.774342 | 17.2 | 3.5 | ND | 6 | 79 | 151 | 75 | 0.1 | 29.7 | 0 |
| **50–22** | Alode | 7.128068 | 4.774547 | 16.2 | 4.5 | 576 | 8.9 | 115 | 175 | 88 | 0.1 | 28.5 | 0 |
| **51–22** | Alode | 7.119213 | 4.774785 | 26.7 | 5.2 | 479 | 7.9 | 103 | 37 | 18 | 0.02 | 28.5 | 1 |
| **52–22** | Alode | 7.119302 | 4.774917 | 26.2 | 4.8 | 453 | 4.8 | 60 | 78 | 39 | 0.04 | 26.9 | 0 |
| **52–23** | Alode | 7.119302 | 4.774917 | 26.2 | 5 | ND | 3.7 | 51 | 76 | 38 | 0.04 | 30.5 | 2 |
| **53–22** | Alode | 7.121002 | 4.774275 | 18.9 | 4.7 | 489 | 5.4 | 67 | 41 | 21 | 0.02 | 26.5 | 0 |
| **54–22** | Alode | 7.121198 | 4.771883 | 17 | 5.6 | 474 | 8.5 | 107 | 23 | 12 | 0.01 | 26.9 | 2 |
| **55–22** | Alode | 7.121875 | 4.773352 | 18.4 | 4.9 | 754 | 7.5 | 99 | 43 | 21 | 0.02 | 28.9 | 0 |
| **56–22** | Alode | 7.123095 | 4.775877 | 26.2 | 5.1 | 536 | 8 | 105 | 22 | 11 | 0.01 | 28.6 | 0 |
| **57–22** | Alode | 7.120873 | 4.7731 | 35.2 | 5.1 | 454 | 7.6 | 94 | 199 | 100 | 0.1 | 26.6 | 0 |
| **58–22** | Alode | 7.121163 | 4.77653 | 16.1 | 5.1 | 438 | 3.9 | 47 | 127 | 63 | 0.1 | 25.4 | 0 |
| **59–22** | Alode | 7.11643 | 4.76728 | 18 | 6.3 | 147 | 3.3 | 43 | 281 | 140 | 0.1 | 27.6 | 5 |
| **60–22** | Alode | 7.114468 | 4.767733 | 8.9 | 6.9 | 117 | 2 | 25 | 388 | 195 | 0.2 | 27.9 | 12 |
| **61–22** | Alode | 7.114897 | 4.765332 | 11 | 6.4 | 231 | 3.3 | 43 | 100 | 50 | 0 | 29 | 3 |
| **62–22** | Alode | 7.128373 | 4.772763 | 11 | 6.3 | 140 | 3.2 | 42 | 269 | 134 | 0.1 | 28.9 | 6 |
| **63–23** | Alode | 7.124539 | 4.77805 | 15 | 5.9 | ND | 3.5 | 48 | 106 | 53 | 0.1 | 30.9 | 30 |
| **64–23** | Alode | 7.123953 | 4.776968 | 16 | 5.6 | ND | 5.9 | 81 | 31 | 15 | 0.01 | 30.9 | 6 |
| **65–23** | Alode | 7.120892 | 4.773005 | 16.1 | 5.3 | ND | 5.4 | 75 | 36 | 18 | 0.02 | 31 | 1 |
| **Minimum** | | | | | 3.5 | 117 | 1.4 | 19 | 20 | 10 | 0 | 25.4 | 0 |
| **Maximum** | | | | | 6.9 | 754 | 8.9 | 115 | 388 | 195 | 0.2 | 33.5 | 62 |
| **Median** | | | | | 5.1 | 438 | 5.5 | 75 | 59 | 30 | 0.03 | 28.9 | 1 |
| **Average** | | | | | 5 | 403 | 5 | 71 | 95 | 48 | 0.04 | 29 | 5 |
| **66–22** | Okochiri | 7.10224 | 4.750622 | 15.2 | 5 | 322 | 4.4 | 57 | 207 | 105 | 0.1 | 28.9 | 2 |
| **67–22** | Okochiri | 7.101903 | 4.75027 | 14.8 | 5.6 | 292 | 4.9 | 66 | 72 | 36 | 0.03 | 30.9 | 8 |
| **67–23** | Okochiri | 7.101903 | 4.75027 | 14.8 | 6.3 | ND | 7 | 93 | 67 | 33 | 0.03 | 29.5 | 14 |
| **68–22** | Okochiri | 7.101417 | 4.750313 | 19.9 | 4.6 | 742 | 5.3 | 69 | 46 | 24 | 0.02 | 28.2 | 1 |
| **68–23** | Okochiri | 7.101417 | 4.750313 | 19.9 | 5.5 | ND | 7.3 | 97 | 27 | 13 | 0.01 | 29.5 | 0 |
| **69–22** | Okochiri | 7.10104 | 4.75044 | 19.9 | 4.7 | 783 | 3.3 | 42 | 67 | 34 | 0.03 | 28.1 | 0 |
| **69–23** | Okochiri | 7.10104 | 4.75044 | 19.9 | 5.2 | ND | 5 | 66 | 26 | 13 | 0.01 | 29.2 | 0 |
| **70–22** | Okochiri | 7.10209 | 4.751638 | 11.7 | 5.6 | 336 | 7.4 | 97 | 79 | 40 | 0.04 | 28.8 | 5 |
| **70–23** | Okochiri | 7.10209 | 4.751638 | 11.7 | 5.7 | ND | 6.4 | 85 | 50 | 25 | 0.02 | 29.4 | 5 |
| **71–22** | Okochiri | 7.100852 | 4.75112 | 8.1 | 4.6 | 728 | 5.2 | 67 | 53 | 26 | 0.02 | 28.3 | 5 |
| **71–23** | Okochiri | 7.100852 | 4.75112 | 8.1 | 5 | ND | 6.5 | 84 | 32 | 16 | 0.01 | 28.3 | 0 |
| **72–22** | Okochiri | 7.100598 | 4.75093 | 0.3 | 4.7 | 513 | 7.5 | 96 | 32 | 16 | 0.01 | 28.1 | 1 |
| **73–22** | Okochiri | 7.100998 | 4.750173 | 11 | 4.8 | 576 | 5.6 | 73 | 39 | 19 | 0.02 | 28.3 | 1 |
| **73–23** | Okochiri | 7.100998 | 4.750173 | 11 | 6.1 | ND | 7.2 | 96 | 31 | 15 | 0.01 | 29.7 | 0 |
| **74–22** | Okochiri | 7.101615 | 4.74998 | 0.1 | 4.6 | 716 | 4.8 | 61 | 41 | 20 | 0.02 | 28 | 1 |
| **74–23** | Okochiri | 7.101615 | 4.74998 | 0.1 | 5.6 | ND | 6.1 | 82 | 28 | 14 | 0.01 | 29.4 | 0 |
| **75–22** | Okochiri | 7.102665 | 4.751113 | 9.3 | 4.4 | 653 | 5.6 | 71 | 75 | 37 | 0.03 | 27.7 | 1 |
| **75–23** | Okochiri | 7.102665 | 4.751113 | 9.3 | 4.5 | ND | 6.7 | 86 | 30 | 15 | 0.01 | 28.1 | 0 |
| **76–22** | Okochiri | 7.102948 | 4.752168 | 8.4 | 4.8 | 273 | 4.4 | 58 | 107 | 54 | 0.1 | 28.9 | 2 |
| **77–22** | Okochiri | 7.108247 | 4.751522 | 7.4 | 4.7 | 395 | 2.5 | 32 | 48 | 24 | 0.02 | 27.5 | 1 |
| **78–22** | Okochiri | 7.102517 | 4.7508 | 0.8 | 4.5 | 734 | 3.2 | 41 | 40 | 20 | 0.02 | 28 | 0 |
| **78–23** | Okochiri | 7.102517 | 4.7508 | 0.8 | 5 | ND | 6.4 | 85 | 21 | 11 | 0.01 | 29.1 | 0 |
| **79–22** | Okochiri | 7.10132 | 4.751203 | 21 | 4.7 | 775 | 6.9 | 96 | 34 | 17 | 0.01 | 32.5 | 0 |
| **79–23** | Okochiri | 7.10132 | 4.751203 | 21 | 5.6 | ND | 6.5 | 84 | 28 | 14 | 0.01 | 28.2 | 0 |
| **ID** | **Site** | **Longitude** | **Latitude** | **Altitude (m)** | **pH** | **Eh (mV)** | **DO (mg/L)** | **DO (%)** | **EC (µS/cm)** | **TDS (mg/L)** | **Salinity (PSU)** | **Temp. (°C)** | **Alkalinity (mg/L)** |
| **80–22** | Okochiri | 7.100848 | 4.751698 | 16.3 | 4.6 | 801 | 6.6 | 85 | 48 | 24 | 0.02 | 28.4 | 0 |
| **81–22** | Okochiri | 7.100475 | 4.751938 | 12.3 | 4.7 | 613 | 6.6 | 85 | 23 | 12 | 0.01 | 27.7 | 0 |
| **82–22** | Okochiri | 7.10029 | 4.752067 | 24.3 | 5.4 | 418 | 4.5 | 59 | 52 | 26 | 0.02 | 28.9 | 4 |
| **82–23** | Okochiri | 7.10029 | 4.752067 | 24.3 | 5.8 | ND | 7.1 | 96 | 18 | 9 | 0.01 | 29.7 | 0 |
| **83–22** | Okochiri | 7.100463 | 4.751511 | 19.4 | 4.7 | 542 | 7.7 | 99 | 121 | 60 | 0.1 | 28.2 | 0.3 |
| **84–22** | Okochiri | 7.100348 | 4.751697 | 16.4 | 4.7 | 507 | 6.2 | 81 | 68 | 33 | 0.03 | 29 | 0 |
| **85–22** | Okochiri | 7.099723 | 4.753902 | 23.4 | 5 | 508 | 7.3 | 95 | 40 | 20 | 0.02 | 29.3 | 1 |
| **85–23** | Okochiri | 7.099723 | 4.753902 | 23.4 | 5.3 | ND | 6.7 | 85 | 17 | 8 | 0.01 | 27.5 | 1 |
| **86–22** | Okochiri | 7.113888 | 4.77924 | 18 | 4.6 | 497 | 8.2 | 102 | 35 | 17 | 0.01 | 26.1 | 1 |
| **87–22** | Okochiri | 7.115607 | 4.775545 | 14.9 | 4.7 | 516 | 8.3 | 102 | 29 | 14 | 0.01 | 26.1 | 0 |
| **88–22** | Okochiri | 7.097222 | 4.765118 | 17.8 | 4.8 | 786 | 6.5 | 81 | 66 | 33 | 0.03 | 26.6 | 1 |
| **88–23** | Okochiri | 7.097222 | 4.765118 | 17.8 | 5.6 | ND | 7.3 | 91 | 17 | 8 | 0.01 | 26.4 | 1 |
| **89–22** | Okochiri | 7.106193 | 4.751553 | 0.1 | 6.1 | 238 | 4.6 | 59 | 98 | 49 | 0.04 | 28.3 | 25 |
| **89–23** | Okochiri | 7.106193 | 4.751553 | 0.1 | 5.1 | ND | 6 | 78 | 35 | 18 | 0.02 | 27.9 | 5 |
| **90–22** | Okochiri | 7.098743 | 4.750856 | 15.9 | 4.7 | 594 | 6.2 | 79 | 178 | 89 | 0.1 | 27.6 | 0.2 |
| **91–22** | Okochiri | 7.105703 | 4.772383 | 15.9 | 4.7 | 487 | 8.4 | 106 | 74 | 37 | 0.03 | 26.9 | 1 |
| **92–22** | Okochiri | 7.09597 | 4.764598 | 8.2 | 5 | 478 | 8.4 | 106 | 219 | 110 | 0.1 | 27 | 1 |
| **93–22** | Okochiri | 7.099103 | 4.749565 | 17.8 | 4.6 | 487 | 7 | 91 | 133 | 66 | 0.1 | 28.3 | 0 |
| **94–22** | Okochiri | 7.099858 | 4.752686 | 14.3 | 4.6 | 769 | 3.1 | 40 | 59 | 29 | 0.03 | 28.8 | 0 |
| **94–23** | Okochiri | 7.099858 | 4.752686 | 14.3 | 5.2 | ND | 4.7 | 64 | 24 | 12 | 0.01 | 30.6 | 0 |
| **95–22** | Okochiri | 7.101355 | 4.749598 | 12.8 | 4.5 | 553 | 8 | 102 | 59 | 30 | 0.03 | 27.8 | 0 |
| **96–22** | Okochiri | 7.103658 | 4.750985 | 13.9 | 4.6 | 535 | 7.4 | 94 | 42 | 21 | 0.02 | 27.4 | 0 |
| **97–22** | Okochiri | 7.115943 | 4.753642 | 24.8 | 4.7 | 516 | 8 | 105 | 136 | 68 | 0.1 | 29.1 | 0 |
| **98–22** | Okochiri | 7.103875 | 4.771045 | 16.6 | 4.8 | 458 | 8.3 | 105 | 55 | 28 | 0.02 | 27.2 | 0 |
| **99–22** | Okochiri | 7.09639 | 4.767717 | 7.3 | 4.9 | 457 | 8.1 | 106 | 132 | 66 | 0.1 | 28.6 | 0 |
| **100–22** | Okochiri | 7.11692 | 4.754807 | 17.1 | 4.8 | 477 | 7 | 87 | 56 | 28 | 0.02 | 26.1 | 0 |
| **101–22** | Okochiri | 7.11262 | 4.75071 | 0.7 | 5.3 | 583 | 6.9 | 87 | 52 | 26 | 0.02 | 26.8 | 0 |
| **102–22** | Okochiri | 7.115581 | 4.742069 | 0.1 | 4.8 | 493 | 7.3 | 93 | 27 | 13 | 0.01 | 27.3 | 0 |
| **103–22** | Okochiri | 7.116902 | 4.749518 | 6.3 | 5.1 | 491 | 8.3 | 108 | 83 | 41 | 0.04 | 28.7 | 1 |
| **104–22** | Okochiri | 7.11887 | 4.751762 | 13.8 | 5.4 | 457 | 8.3 | 108 | 42 | 21 | 0.02 | 28.8 | 0 |
| **105–22** | Okochiri | 7.116783 | 4.747585 | 0.5 | 5.5 | 451 | 7.9 | 99 | 26 | 13 | 0.01 | 26.6 | 0.3 |
| **106–22** | Okochiri | 7.109768 | 4.749522 | 23.2 | 4.8 | 781 | 5.7 | 74 | 52 | 26 | 0.02 | 28.6 | 0 |
| **107–22** | Okochiri | 7.109265 | 4.746812 | 8.7 | 4.8 | 539 | 7.9 | 100 | 24 | 12 | 0.01 | 27.4 | 0 |
| **108–22** | Okochiri | 7.100618 | 4.750235 | 12.8 | 5 | 737 | 6.2 | 80 | 45 | 22 | 0.02 | 28.1 | 1 |
| **108–23** | Okochiri | 7.100618 | 4.750235 | 12.8 | 5.6 | ND | 7.3 | 93 | 24 | 12 | 0.01 | 27.2 | 1 |
| **109–23** | Okochiri | 7.100833 | 4.751111 | 10.2 | 4.5 | ND | 7.2 | 94 | 18 | 9 | 0.01 | 28.3 | 0 |
| **110–23** | Okochiri | 7.100833 | 4.750556 | 10.1 | 4.5 | ND | 4.8 | 62 | 32 | 16 | 0.01 | 28.4 | 0 |
| **111–23** | Okochiri | 7.100028 | 4.751111 | 10 | 5.6 | ND | 7.2 | 94 | 17 | 9 | 0.01 | 28.5 | 0 |
| **112–23** | Okochiri | 7.100028 | 4.750557 | 9 | 5.4 | ND | 7.3 | 95 | 16 | 8 | 0.01 | 28.5 | 0 |
| **113–23** | Okochiri | 7.099083 | 4.750546 | 6 | 5.6 | ND | 6.3 | 83 | 64 | 32 | 0.03 | 28.3 | 14 |
| **114–23** | Okochiri | 7.105556 | 4.751389 | 8 | 5.2 | ND | 5.3 | 70 | 27 | 13 | 0.01 | 29 | 0 |
| **115–23** | Okochiri | 7.097785 | 4.657397 | 13 | 4.7 | ND | 4.4 | 58 | 20 | 10 | 0.01 | 29 | 0 |
| **Minimum** | | | | | 4.4 | 238 | 2.5 | 32 | 16 | 8 | 0.01 | 26.1 | 0 |
| **Maximum** | | | | | 6.2 | 801 | 8.4 | 108 | 219 | 110 | 0.1 | 32.5 | 25 |
| **Median** | | | | | 4.8 | 516 | 6.7 | 85 | 42 | 21 | 0.02 | 28.3 | 0 |
| **Average** | | | | | 5 | 549 | 6.4 | 83 | 56 | 28 | 0.03 | 28 | 1.6 |
| **All data** | | | | | | | | | | | | | |
| **Minimum** | | | | | 3.5 | 113 | 0.7 | 9 | 16 | 8 | 0.01 | 25.1 | 0 |
| **Maximum** | | | | | 6.9 | 801 | 8.9 | 115 | 852 | 427 | 0.4 | 33.5 | 100 |
| **Median** | | | | | 5 | 489 | 6.2 | 81 | 67 | 33 | 0.03 | 28.5 | 0 |
| **Average** | | | | | 5 | 485 | 5.9 | 76 | 120 | 60 | 0.1 | 28.5 | 5 |
| **REF1** | Alode | 7.11569 | 4.775232 | 14 | 4 | 491 | 7.6 | 103 | 24 | 12 | 0.01 | 30.9 | 0 |
| **REF2** | Alode | 7.114142 | 4.779242 | 14 | 4.3 | 516 | 7.5 | 101 | 17 | 9 | 0.01 | 30.8 | 0 |
| **REF3** | Okochiri | 7.114741 | 4.747028 | 10.4 | 4.6 | 451 | 6.6 | 84 | 19 | 9 | 0.01 | 27 | 2 |
| **REF4** | Okrika | 7.079138 | 4.738417 | 1.1 | 5.1 | 450 | 7.9 | 100 | 31 | 15 | 0.01 | 27.7 | 1 |
| **REF5** | Ogale | 7.14101 | 4.774265 | 18.2 | 5 | 641 | 8.2 | 107 | 69 | 32 | 0.03 | 28.4 | 0 |

**Table 2S: Chemical measurements and hydrochemical ratios of groundwater in the study area (2022 and 2023)**

| **ID** | **Site** | **NO₃ (mg/L)** | **NO₂ (mg/L)** | **F (mg/L)** | **Cl (mg/L)** | **SO₄² (mg/L)** | **Ca (mg/L)** | **Na (mg/L)** | **K (mg/L)** | **Mg (mg/L)** | **Si (mg/L)** | **Sr (mg/L)** | **Fe (mg/L)** | **Mn (mg/L)** | **DOC (mg/L)** | **NO₃^-^/Cl^-^** |
| --- | --- | --- | --- | --- | --- | --- | --- | --- | --- | --- | --- | --- | --- | --- | --- | --- |
| **1–22** | Alesa | 142 | 0.3 | 0.2 | 49 | < 0.01 | 27 | 32 | 19 | 5 | 2 | 0.1 | <0.01 | 0.1 | 21 | 2.9 |
| **1–23** | Alesa | 106 | <0.01 | <0.01 | 41 | 21 | 27 | 33 | 18 | 5 | 2 | 0.1 | <0.01 | 0.1 | 31 | 2.6 |
| **2–22** | Alesa | 39 | 1 | 0.2 | 17 | 23 | 13 | 14 | 12 | 4 | 1 | 0.01 | <0.01 | <0.02 | 20 | 2.3 |
| **2–23** | Alesa | 34 | 1 | 0.2 | 17 | 23 | 12 | 15 | 10 | 3 | 3 | 0.01 | <0.01 | <0.02 | 36 | 2 |
| **3–22** | Alesa | 83 | <0.01 | 0.2 | 30 | 4 | 10 | 25 | 4 | 2 | 1 | 0.03 | <0.01 | 0.1 | 31 | 2.8 |
| **3–23** | Alesa | 83 | <0.01 | 0.3 | 27 | 4 | 8 | 23 | 2 | 2 | 3 | 0.03 | <0.01 | 0.1 | 27 | 3.1 |
| **4–22** | Alesa | 19 | <0.01 | 0.1 | 10 | 2 | 12 | 10 | 1 | 3 | 1 | 0.02 | <0.01 | 0.04 | 33 | 1.9 |
| **4–23** | Alesa | 17 | <0.01 | <0.01 | 9 | 1 | 3 | 9 | 1 | 1 | 3 | 0.01 | <0.01 | 0.02 | 37 | 1.9 |
| **5–22** | Alesa | 90 | <0.01 | 0.2 | 27 | 5 | 12 | 27 | 7 | 3 | 1 | 0.04 | <0.01 | 0.1 | 17 | 3.3 |
| **5–23** | Alesa | 73 | <0.01 | <0.01 | 24 | 5 | 10 | 22 | 6 | 2 | 2 | 0.04 | <0.01 | 0.1 | 38 | 3 |
| **ID** | **Site** | **NO₃ (mg/L)** | **NO₂ (mg/L)** | **F (mg/L)** | **Cl (mg/L)** | **SO₄² (mg/L)** | **Ca (mg/L)** | **Na (mg/L)** | **K (mg/L)** | **Mg (mg/L)** | **Si (mg/L)** | **Sr (mg/L)** | **Fe (mg/L)** | **Mn (mg/L)** | **DOC (mg/L)** | **NO₃^-^/Cl^-^** |
| **6–22** | Alesa | 58 | <0.01 | 0.2 | 18 | 14 | 11 | 15 | 11 | 3 | 1 | 0.02 | <0.01 | 0.02 | 41 | 3.2 |
| **6–23** | Alesa | 53 | <0.01 | 0.1 | 18 | 13 | 10 | 16 | 9 | 2 | 3 | 0.02 | <0.01 | 0.02 | 37 | 2.9 |
| **7–22** | Alesa | 99 | <0.01 | 0.2 | 40 | 21 | 18 | 29 | 14 | 4 | 1 | 0.1 | 0.04 | 0.2 | 32 | 2.5 |
| **7–23** | Alesa | 97 | <0.01 | 0.1 | 38 | 18 | 16 | 29 | 12 | 3 | 3 | 0.1 | <0.01 | 0.2 | 37 | 2.6 |
| **8–22** | Alesa | 33 | <0.01 | 0.2 | 10 | 14 | 7 | 10 | 8 | 2 | 1 | 0.01 | <0.01 | 0.03 | 26 | 3.3 |
| **8–23** | Alesa | 32 | <0.01 | 0.2 | 11 | 14 | 7 | 9 | 7 | 2 | 3 | 0.01 | <0.01 | 0.02 | 42 | 2.9 |
| **9–22** | Alesa | 68 | <0.01 | <0.01 | 24 | 13 | 51 | 18 | 14 | 8 | 2 | 0.1 | <0.01 | 0.3 | 24 | 2.8 |
| **9–23** | Alesa | 66 | <0.01 | <0.01 | 24 | 12 | 16 | 20 | 12 | 4 | 2 | 0.03 | <0.01 | 0.1 | 35 | 2.8 |
| **10–22** | Alesa | 18 | <0.01 | 0.2 | 5 | 2 | 1 | 7 | 1 | 0.3 | 1 | 0.01 | <0.01 | 0.1 | 15 | 3.6 |
| **10–23** | Alesa | 22 | <0.01 | <0.01 | 6 | 1 | 2 | 7 | 1 | 0.3 | 3 | 0.01 | <0.01 | 0.1 | 32 | 3.7 |
| **Minimum** | | 17 | <0.01 | <0.01 | 5 | 1 | 1 | 7 | 1 | 0.3 | 1 | 0.01 | <0.01 | <0.02 | 15 | 1.9 |
| **Maximum** | | 142 | 1 | 0.3 | 49 | 23 | 51 | 33 | 19 | 8 | 3 | 0.1 | 0.04 | 0.3 | 42 | 3.7 |
| **Median** | | 62 | 1 | 0.2 | 21 | 13 | 12 | 17 | 9 | 3 | 2 | 0.03 | 0.04 | 0.1 | 32 | 2.9 |
| **Average** | | 62 | 0.8 | 0.2 | 22 | 11 | 14 | 19 | 9 | 3 | 2 | 0.04 | 0.04 | 0.1 | 31 | 2.8 |
| **11–22** | Ogale | <0.01 | <0.01 | <0.01 | 11 | 3 | 0.3 | 11 | 0.3 | 0.1 | 1 | 0.001 | 2 | <0.02 | 16 | <0.01 |
| **12–22** | Ogale | 2 | 0.2 | <0.01 | 5 | 3 | 13 | 4 | 1 | 1 | 0.3 | 0.01 | 50 | 0.1 | 49 | 0.4 |
| **13–22** | Ogale | 3 | <0.01 | 0.1 | 7 | 31 | 15 | 8 | 7 | 3 | 0.4 | 0.04 | 0.03 | 0.1 | 24 | 0.4 |
| **13–23** | Ogale | <0.01 | <0.01 | 0.1 | 10 | 7 | 12 | 4 | 7 | 1 | 1 | 0.01 | 46 | 0.1 | 30 | <0.01 |
| **14–22** | Ogale | 93 | 0.2 | 0.2 | 57 | 17 | 14 | 50 | 19 | 3 | 0.4 | 0.04 | 0.02 | 0.4 | 32 | 1.6 |
| **15–22** | Ogale | 12 | <0.01 | <0.01 | 4 | 4 | 0.4 | 3 | 1 | 0.1 | 1 | 0.002 | 3 | 0.02 | 28 | 3 |
| **15–23** | Ogale | 64 | <0.01 | <0.01 | 49 | 18 | 10 | 39 | 18 | 2 | 1 | 0.03 | 0.03 | 0.2 | 27 | 1.3 |
| **16–22** | Ogale | <0.01 | <0.01 | <0.01 | 6 | 2 | 0.4 | 5 | 0.2 | <0.01 | 1 | 0.001 | 2 | <0.02 | 27 | <0.01 |
| **16–23** | Ogale | <0.01 | <0.01 | <0.01 | 5 | 3 | 0.2 | 4 | 0.3 | 0.1 | 4 | <0.001 | 2 | 0.02 | 32 | <0.01 |
| **17–22** | Ogale | 211 | <0.01 | 0.4 | 66 | 80 | 42 | 56 | 59 | 14 | 1 | 0.1 | <0.02 | 0.2 | 47 | 3.2 |
| **17–23** | Ogale | <0.01 | <0.01 | 0.3 | 2 | 3 | 1 | 1 | 1 | 0.2 | 6 | <0.001 | 0.02 | 0.02 | 5 | <0.01 |
| **18–22** | Ogale | 12 | 0.2 | 0.1 | 8 | 2 | 2 | 11 | 1 | 1 | 1 | 0.02 | 0.04 | 0.2 | 19 | 1.5 |
| **19–22** | Ogale | 2 | <0.01 | 0.2 | 3 | 2 | 0.4 | 1 | 0.3 | 0.1 | 1 | 0.003 | 1 | 0.1 | 15 | 0.7 |
| **19–23** | Ogale | <0.01 | <0.01 | <0.01 | 8 | 2 | 0.4 | 5 | 0.1 | 0.2 | 4 | <0.001 | 1 | 0.04 | 30 | <0.01 |
| **20–22** | Ogale | 124 | <0.01 | 0.3 | 30 | 2 | 13 | 30 | 5 | 3 | 1 | 0.05 | 0.03 | 1 | 17 | 4.1 |
| **20–23** | Ogale | <0.01 | <0.01 | <0.01 | 3 | 2 | 0.3 | 1.4 | 0.1 | 0.1 | 3 | <0.001 | 1 | 0.04 | 31 | <0.01 |
| **21–22** | Ogale | 20 | <0.01 | 0.2 | 5 | 2 | 3 | 6 | 2 | 1 | 1 | 0.01 | <0.01 | 0.1 | 17 | 4 |
| **21–23** | Ogale | 81 | <0.01 | 0.1 | 21 | 1 | 7 | 19 | 4 | 2 | 3 | 0.03 | <0.01 | 1 | 36 | 3.9 |
| **22–22** | Ogale | <0.01 | <0.01 | <0.01 | 5 | 2 | 0.3 | 3 | 0.3 | 0.1 | 1 | 0.001 | 4 | 0.02 | 9 | <0.01 |
| **23–22** | Ogale | <0.01 | <0.01 | <0.01 | 3 | 1 | 1 | 2 | 1 | 0.1 | 1 | 0.001 | 2 | 0.02 | 14 | <0.01 |
| **23–23** | Ogale | <0.01 | <0.01 | <0.01 | 6 | 1 | 0.2 | 3 | 0.1 | 0.1 | 3 | <0.001 | 4 | 0.02 | 27 | <0.01 |
| **24–22** | Ogale | 29 | <0.01 | 0.2 | 6 | 2 | 0.4 | 1 | 0.2 | 0.1 | 1 | 0.001 | 0.1 | 0.02 | 16 | 4.8 |
| **24–23** | Ogale | <0.01 | <0.01 | <0.01 | 6 | 2 | 0.2 | 5 | 0.1 | <0.01 | 3 | <0.001 | 1 | <0.02 | 26 | < 0.001 |
| **25–22** | Ogale | 29 | <0.01 | 0.2 | 7 | 2 | 3 | 10 | 0.4 | 1 | 1 | 0.02 | <0.01 | 0.1 | 14 | 4.1 |
| **25–23** | Ogale | 28 | <0.01 | 0.1 | 7 | 1 | 2 | 11 | 0.4 | 0.4 | 3 | 0.01 | <0.01 | 0.1 | 36 | 4 |
| **26–22** | Ogale | 88 | <0.01 | 0.3 | 24 | 2 | 7 | 25 | 1 | 3 | 2 | 0.1 | 0.02 | 1 | 11 | 3.7 |
| **26–23** | Ogale | 22 | <0.01 | <0.01 | 6 | <0.01 | 2 | 9 | 0.2 | 0.3 | 3 | 0.01 | 0.02 | 0.1 | 35 | 3.7 |
| **27–22** | Ogale | 51 | 0.2 | 0.3 | 36 | 21 | 6 | 29 | 10 | 2 | 1 | <0.001 | 0.02 | 0.1 | 25 | 1.4 |
| **27–23** | Ogale | 62 | <0.01 | 0.1 | 19 | 1 | 5 | 17 | 2 | 2 | 3 | 0.03 | <0.01 | 1 | 35 | 3.3 |
| **Minimum** | | <0.01 | <0.01 | <0.01 | 2 | <0.01 | 0.2 | 1 | 0.1 | <0.01 | 0.3 | <0.001 | <0.01 | <0.02 | 5 | <0.001 |
| **Maximum** | | 211 | 0.2 | 0.4 | 66 | 80 | 42 | 56 | 59 | 14 | 6 | 0.1 | 50 | 1 | 49 | 4.8 |
| **Median** | | 29 | 0.2 | 0.2 | 7 | 2 | 2 | 6 | 1 | 1 | 1 | 0.01 | 1 | 0.1 | 27 | 3.3 |
| **Average** | | 57 | 0.2 | 0.2 | 16 | 10 | 7 | 14 | 7 | 2 | 2 | 0.03 | 7 | 0.3 | 25 | 2.7 |
| **28–22** | Ebubu | 2 | <0.01 | <0.01 | 1 | 2 | 1 | 1 | 0.2 | 0.1 | 1 | 0.004 | <0.01 | <0.02 | 25 | 1.4 |
| **29–22** | Ebubu | <0.01 | <0.01 | 0.3 | 1 | 8 | 15 | 26 | 7 | 3 | 1 | 0.1 | 0.4 | 0.4 | 32 | <0.01 |
| **30–22** | Ebubu | 13 | <0.01 | 0.2 | 3 | 2 | 3 | 5 | <0.01 | 1 | 2 | 0.01 | 0.05 | 0.2 | 26 | 3.9 |
| **31–22** | Ebubu | 2 | <0.01 | <0.01 | 1 | 2 | 0.3 | 1 | 0.2 | 0.1 | 1 | 0.001 | <0.01 | <0.02 | 35 | 1.4 |
| **31–23** | Ebubu | 15 | <0.01 | <0.01 | 4 | <0.01 | 1 | 6 | 0.2 | 0.3 | 3 | 0.01 | <0.01 | 0.1 | 31 | 3.8 |
| **32–22** | Ebubu | 51 | <0.01 | 0.2 | 12 | 1 | 12 | 12 | 2 | 3 | 2 | 0.03 | 0.2 | 2 | 31 | 4.3 |
| **33–22** | Ebubu | 8 | <0.01 | <0.01 | 3 | 2 | 1 | 3.4 | 0.3 | 0.1 | 1 | 0.003 | 0.02 | 0.02 | 34 | 2.7 |
| **33–23** | Ebubu | 44 | <0.01 | 0.1 | 9 | 1 | 3 | 11 | 2 | 1 | 3 | 0.01 | 0.03 | 1 | 32 | 4.9 |
| **34–22** | Ebubu | 120 | 0.2 | 0.2 | 40 | 2 | 31 | 27 | 16 | 8 | 1 | 0.1 | 0.05 | 0.1 | 34 | 3 |
| **34–23** | Ebubu | 10 | 1 | 0.2 | 6 | 6 | 1 | 5 | 0.1 | 0.2 | 3 | <0.001 | <0.02 | 0.02 | 27 | 1.7 |
| **35–22** | Ebubu | 15 | 0.2 | 0.2 | 4 | 4 | 8 | 5 | 0.3 | 2 | 1 | 0.02 | 0.1 | 0.1 | 19 | 3.8 |
| **35–23** | Ebubu | 148 | <0.01 | 0.2 | 42 | 26 | 29 | 27 | 14 | 7 | 3 | 0.1 | 0.02 | 0.1 | 33 | 3.5 |
| **36–22** | Ebubu | 11 | <0.01 | <0.01 | 4 | 6 | 4 | 3 | 3 | 1 | 1 | 0.004 | 0.02 | 0.02 | 25 | 2.8 |
| **36–23** | Ebubu | 3 | <0.01 | <0.01 | 4 | 1 | 1 | 5 | 0.1 | 0.2 | 3 | <0.001 | <0.01 | 0.02 | 27 | 0.8 |
| **37–22** | Ebubu | 2 | <0.01 | <0.01 | 1 | 2 | 1 | 1 | 0.2 | 0.1 | 1 | 0.002 | <0.01 | <0.02 | 35 | 1.4 |
| **38–22** | Ebubu | 19 | < 0.01 | 0.2 | 7 | 2 | 1 | 8 | 0.4 | 1 | 1 | 0.01 | <0.01 | 0.2 | 47 | 2.6 |
| **38–23** | Ebubu | 25 | <0.01 | <0.01 | 8 | 1 | 1 | 11 | 0.3 | 1 | 3 | 0.01 | <0.01 | 0.2 | 39 | 3.1 |
| **39–22** | Ebubu | 11 | <0.01 | 0.2 | 3 | 2 | 3 | 5 | 0.3 | 1 | 1 | 0.01 | 0.02 | 0.1 | 32 | 3.2 |
| **39–23** | Ebubu | 28 | <0.01 | 0.1 | 8 | 3 | 1 | 11 | 0.3 | 1 | 3 | 0.01 | <0.01 | 0.2 | 39 | 3.3 |
| **Minimum** | | <0.01 | <0.01 | <0.01 | 1 | <0.01 | 0.3 | 1 | <0.01 | 0.1 | 1 | <0.001 | <0.01 | <0.02 | 19 | <0.01 |
| **Maximum** | | 148 | 1 | 0.3 | 42 | 26 | 31 | 27 | 16 | 8 | 3 | 0.1 | 0.4 | 2 | 47 | 4.9 |
| **Median** | | 14 | 0.2 | 0.2 | 4 | 2 | 1 | 5 | 0.3 | 1 | 1 | 0.01 | 0.04 | 0.1 | 32 | 3 |
| **Average** | | 34 | 0.5 | 0.1 | 10 | 5 | 7 | 10 | 3 | 2 | 2 | 0.03 | 0.1 | 0.4 | 32 | 2.9 |
| **40–22** | Alode | <0.01 | <0.01 | <0.01 | 1 | 1 | 2 | 10 | 1 | 0.4 | 1 | 0.01 | <0.01 | 0.2 | 47 | <0.01 |
| **40–23** | Alode | <0.01 | <0.01 | <0.01 | 3 | 1 | 10 | 1 | 1 | 1 | 3 | 0.03 | 0.3 | 0.03 | 35 | <0.01 |
| **41–22** | Alode | 0.8 | <0.01 | <0.01 | 4 | 2 | 0.4 | 4 | 0.2 | 0.1 | 1 | 0.001 | 0.2 | 0.02 | 17 | 0.2 |
| **ID** | **Site** | **NO₃ (mg/L)** | **NO₂ (mg/L)** | **F (mg/L)** | **Cl (mg/L)** | **SO₄² (mg/L)** | **Ca (mg/L)** | **Na (mg/L)** | **K (mg/L)** | **Mg (mg/L)** | **Si (mg/L)** | **Sr (mg/L)** | **Fe (mg/L)** | **Mn (mg/L)** | **DOC (mg/L)** | **NO₃^-^/Cl^-^** |
| **42–22** | Alode | <0.01 | <0.01 | <0.01 | 7 | 2 | 1 | 5 | 1 | 0.2 | 1 | 0.002 | 2 | <0.02 | 26 | <0.01 |
| **42–23** | Alode | <0.01 | <0.01 | <0.01 | 10 | 2 | 1 | 7 | 0.2 | 0.2 | 3 | <0.001 | 2.4 | <0.02 | 26 | <0.01 |
| **43–22** | Alode | 2 | <0.01 | <0.01 | 10 | 33 | 27 | 6 | 0.4 | 4 | 1 | 0.1 | 19 | 0.3 | 26 | 0.2 |
| **44–22** | Alode | 13 | <0.01 | <0.01 | 9 | 2 | 3 | 9 | 0.4 | 0.4 | 1 | 0.01 | 0.02 | 0.02 | 17 | 1.4 |
| **44–23** | Alode | 1 | 0.2 | 0.4 | 9 | 4 | 2 | 9 | 0.3 | 0.3 | 3 | 0.01 | <0.02 | <0.02 | 28 | 0.1 |
| **45–22** | Alode | <0.01 | <0.01 | 1 | 1 | 2 | 2 | 1 | 1 | 0.3 | 1 | 0.01 | 0.2 | 0.03 | 34 | <0.01 |
| **45–23** | Alode | <0.01 | <0.01 | <0.01 | 2 | 4 | 1.3 | 5 | 0.4 | 0.3 | 4 | <0.001 | 0.3 | 0.02 | 32 | <0.01 |
| **46–22** | Alode | <0.01 | <0.01 | 3 | 1 | 1 | 2 | 1 | 1 | 0.3 | 1 | 0.01 | 1 | 0.03 | 33 | <0.01 |
| **46–23** | Alode | <0.01 | <0.01 | <0.01 | 2 | 1 | 2 | 1 | 0.3 | 0.3 | 4 | 0.01 | 0.4 | 0.03 | 34 | <0.01 |
| **47–22** | Alode | <0.01 | <0.01 | 2 | 1 | 2 | 1 | 1 | 1 | 0.2 | 1 | 0.004 | 0.3 | 0.03 | 26 | <0.01 |
| **47–23** | Alode | <0.01 | <0.01 | <0.01 | 1 | 1 | 1 | 1 | 0.4 | 0.2 | 4 | <0.001 | 1 | 0.02 | 34 | <0.01 |
| **48–22** | Alode | <0.01 | <0.01 | <0.01 | 1 | 2 | 1 | 1 | 1 | 0.2 | 1 | 0.004 | 1 | 0.03 | 22 | <0.01 |
| **48–23** | Alode | <0.01 | <0.01 | <0.01 | 2 | 1 | 1 | 1 | 0.3 | 0.2 | 4 | <0.001 | 1 | 0.04 | 35 | <0.01 |
| **49–22** | Alode | <0.01 | <0.01 | <0.01 | 1 | 2 | 1 | 1 | 1 | 0.3 | 1 | 0.004 | 0.1 | 0.03 | 33 | <0.01 |
| **49–23** | Alode | 55 | <0.01 | <0.01 | 12 | 2 | 3 | 16 | 2 | 1 | 3 | 0.02 | 0.03 | 1 | 42 | 4.6 |
| **50–22** | Alode | 50 | <0.01 | 0.2 | 11 | 2 | 3 | 15 | 2 | 1 | 1 | 0.02 | 0.1 | 0.3 | 10 | 4.5 |
| **51–22** | Alode | 4 | <0.01 | <0.01 | 2 | 2 | 1 | 2 | 1 | 0.1 | 2 | 0.003 | 0.1 | 0.03 | 25 | 2 |
| **52–22** | Alode | 2 | <0.01 | 0.2 | 6 | 17 | 2 | 5 | 0.3 | 0.3 | 1 | 0.01 | 6 | 0.1 | 16 | 0.3 |
| **52–23** | Alode | <0.01 | <0.01 | <0.01 | 9 | 17 | 1 | 10 | 0.1 | 0.2 | 2 | <0.001 | 7 | 0.02 | 30 | <0.01 |
| **53–22** | Alode | 1 | <0.01 | <0.01 | 1 | 1 | 2 | 1 | 1 | 0.4 | 1 | 0.01 | 0.2 | 0.03 | 24 | 1 |
| **54–22** | Alode | 2 | <0.01 | <0.01 | 2 | 2 | 2 | 1 | 0.4 | 0.1 | 1 | 0.002 | <0.01 | <0.02 | 20 | 1 |
| **55–22** | Alode | 7 | <0.01 | <0.01 | 3 | 2 | 1 | 4 | 1 | 0.2 | 1 | 0.004 | <0.01 | 0.02 | 29 | 2.3 |
| **56–22** | Alode | 2 | <0.01 | <0.01 | 2 | 1 | 1 | 1 | 0.3 | 0.1 | 1 | 0.002 | 0.1 | 0.02 | 16 | 0.7 |
| **57–22** | Alode | 2 | <0.01 | <0.01 | 2 | 2 | 1 | 1 | 0.4 | 0.1 | 1 | 0.001 | 1 | <0.02 | 16 | 1 |
| **58–22** | Alode | 1 | <0.01 | <0.01 | 6 | 9 | 2 | 6 | 1 | 0.3 | 1 | 0.01 | 3 | 0.03 | 23 | 0.2 |
| **59–22** | Alode | 2 | <0.01 | <0.01 | 5 | 2 | 7 | 2 | 0.3 | 0.4 | 1 | <0.001 | 32 | <0.02 | 27 | 0.3 |
| **60–22** | Alode | 1 | 2 | 0.3 | 5 | 1 | 40 | 2 | 4 | 1 | 8 | 1 | 1 | 0.1 | 32 | 0.2 |
| **61–22** | Alode | 2 | <0.01 | 0.3 | 2.4 | <0.01 | 8 | 2 | 2 | 4 | 4 | 0.1 | 0.2 | 0.3 | 28 | 0.8 |
| **62–22** | Alode | <0.01 | <0.01 | 0.3 | 3 | <0.01 | 5 | 2 | 2 | 0.3 | 2 | <0.001 | 21 | 0.1 | 54 | <0.01 |
| **63–23** | Alode | <0.01 | <0.01 | <0.01 | 9 | 6 | 1 | 3 | 0.3 | 0.2 | 4 | <0.001 | 3 | 0.02 | 26 | <0.01 |
| **64–23** | Alode | <0.01 | <0.01 | <0.01 | 3 | 2 | 3 | 6 | 0.1 | 1 | 1 | <0.001 | 21 | 0.1 | 29 | <0.01 |
| **65–23** | Alode | 2 | <0.01 | 0.3 | 3 | 3 | 1 | 3 | 0.2 | 0.1 | 3 | <0.001 | 0.4 | 0.01 | 18 | 0.7 |
| **Minimum** | | <0.01 | <0.01 | <0.01 | 1 | <0.01 | 0.4 | 1 | 0.1 | 0.1 | 1 | <0.001 | <0.01 | <0.02 | 10 | <0.01 |
| **Maximum** | | 55 | 2 | 3 | 12 | 33 | 40 | 16 | 4 | 4 | 8 | 1 | 32 | 1 | 54 | 4.6 |
| **Median** | | 2 | 1 | 0.3 | 3 | 2 | 2 | 2 | 0.4 | 0.3 | 1 | 0.01 | 1 | 0.03 | 27 | 0.8 |
| **Average** | | 10 | 1 | 1 | 4.4 | 5 | 5 | 4.4 | 11 | 1 | 2 | 0.1 | 5 | 0.1 | 28 | 1.3 |
| **66–22** | Okochiri | 3 | <0.01 | <0.01 | 3 | 6 | 1 | 7 | 0.4 | 0.1 | 1 | 0.002 | 0.3 | 0.1 | 33 | 1 |
| **67–22** | Okochiri | 2 | <0.01 | <0.01 | 3 | 12 | 1 | 15 | 0.4 | 0.1 | 1 | 0.002 | 0.2 | 0.03 | 27 | 0.7 |
| **67–23** | Okochiri | <0.01 | <0.01 | 0.2 | 3 | 12 | 2 | 14 | 0.2 | 0.1 | 3 | <0.001 | 0.2 | 0.03 | ND | <0.01 |
| **68–22** | Okochiri | 3 | <0.01 | <0.01 | 3 | 4 | 2 | 4 | 1 | 0.2 | 2 | 0.004 | 0.02 | 0.1 | 26 | 1 |
| **68–23** | Okochiri | 1 | <0.01 | <0.01 | 3 | 5 | 2 | 4 | 0.3 | 0.2 | 5 | <0.001 | <0.01 | 0.04 | ND | 0.3 |
| **69–22** | Okochiri | 3 | <0.01 | <0.01 | 3 | 3 | 2 | 3 | 1 | 0.2 | 2 | 0.003 | <0.01 | 0.1 | 24 | 1 |
| **69–23** | Okochiri | 2 | <0.01 | <0.01 | 3 | 2 | 0.4 | 3 | 0.4 | 0.2 | 5 | <0.001 | <0.01 | 0.04 | ND | 0.7 |
| **70–22** | Okochiri | 2 | <0.01 | <0.01 | 4 | 11 | 1 | 10 | 0.3 | 0.2 | 1 | 0.003 | 1 | 0.1 | 22 | 0.5 |
| **70–23** | Okochiri | <0.01 | 0.2 | <0.01 | 3 | 11 | 1 | 10 | 0.2 | 0.2 | 3 | <0.001 | 1 | 0.1 | 3 | <0.01 |
| **71–22** | Okochiri | 3 | <0.01 | <0.01 | 3 | 3 | 1 | 2 | 0.3 | 0.2 | 1 | 0.003 | <0.01 | 0.1 | 23 | 1 |
| **71–23** | Okochiri | 2 | <0.01 | 1.3 | 3 | 3 | 1 | 2 | 0.2 | 0.2 | 4 | <0.001 | <0.01 | 0.04 | ND | 0.7 |
| **72–22** | Okochiri | 2 | <0.01 | <0.01 | 2 | 2 | 1 | 1 | 0.4 | 0.2 | 2 | 0.002 | 0.02 | 0.04 | 29 | 1 |
| **73–22** | Okochiri | 4 | <0.01 | <0.01 | 2 | 2 | 1 | 3 | 1 | 0.2 | 2 | 0.003 | 0.03 | 0.04 | 23 | 2 |
| **73–23** | Okochiri | 3 | <0.01 | <0.01 | 3 | 2 | 1 | 3 | 0.4 | 0.2 | 5 | < 0.001 | <0.02 | 0.03 | 2 | 1 |
| **74–22** | Okochiri | 2 | <0.01 | <0.01 | 2 | 4 | 2 | 4 | 0.2 | 0.3 | 3 | 0.01 | 0.02 | 0.1 | 22 | 1 |
| **74–23** | Okochiri | <0.01 | <0.01 | <0.01 | 2 | 5 | 1 | 4 | 0.3 | 0.2 | 4 | <0.001 | 0.02 | 0.04 | 3 | <0.01 |
| **75–22** | Okochiri | <0.01 | <0.01 | <0.01 | 1.4 | 2 | 1 | 2 | 0.4 | 0.3 | 1 | 0.01 | 0.03 | 1 | 28 | <0.01 |
| **75–23** | Okochiri | <0.01 | <0.01 | <0.01 | 2 | 1 | 1 | 1.4 | 0.2 | 0.2 | 4 | <0.001 | 0.1 | 0.4 | 33 | <0.01 |
| **76–22** | Okochiri | <0.01 | <0.01 | <0.01 | 2 | 5 | 1 | 4 | 0.4 | 0.4 | 2 | 0.01 | 4 | 0.3 | 25 | <0.01 |
| **77–22** | Okochiri | <0.01 | <0.01 | <0.01 | 4 | 1 | 1 | 2 | 0.2 | 0.2 | 1 | 0.003 | 2 | 0.1 | 20 | <0.01 |
| **78–22** | Okochiri | 1 | <0.01 | <0.01 | 1 | 1 | 1 | 1 | 1 | 0.2 | 2 | 0.003 | <0.01 | 0.2 | 22 | 1 |
| **78–23** | Okochiri | 0 | <0.01 | <0.01 | 2 | 1 | 1 | 1 | 0.4 | 0.2 | 5 | <0.001 | <0.01 | 0.3 | 4 | <0.01 |
| **79–22** | Okochiri | 3 | <0.01 | <0.01 | 2 | 3 | 1 | 2 | 1 | 0.3 | 2 | 0.01 | <0.01 | 0.1 | 15 | 1.5 |
| **79–23** | Okochiri | 2 | <0.01 | <0.01 | 3 | 2 | 1 | 2 | 0.4 | 0.2 | 5 | <0.001 | <0.01 | 0.1 | ND | 0.7 |
| **80–22** | Okochiri | 2 | <0.01 | <0.01 | 2 | 3 | 1 | 2 | 0.3 | 0.2 | 1 | 0.003 | <0.01 | 0.1 | 14 | 1.2 |
| **81–22** | Okochiri | 2 | <0.01 | <0.01 | 2 | 1 | 1 | 1 | 0.4 | 0.2 | 1 | 0.002 | <0.01 | 0.04 | 30 | 1 |
| **82–22** | Okochiri | <0.01 | <0.01 | <0.01 | 4 | 5 | 0.3 | 9.4 | 0.2 | <0.01 | 1 | <0.001 | 1 | 0.01 | 33 | <0.01 |
| **82–23** | Okochiri | 1 | <0.01 | <0.01 | 2 | 2 | 0.4 | 2 | 0.2 | 0.2 | 4 | <0.001 | 0.02 | 0.04 | ND | 0.5 |
| **83–22** | Okochiri | 2 | <0.01 | 1 | 2 | 2 | 1 | 1 | 0.2 | 0.2 | 1 | 0.002 | 0.02 | 0.04 | 17 | 1 |
| **84–22** | Okochiri | 3 | <0.01 | <0.01 | 2 | 2 | 1 | 1 | 0.4 | 0.3 | 2 | 0.004 | 0.02 | 0.1 | 34 | 1.5 |
| **85–22** | Okochiri | <0.01 | <0.01 | <0.01 | 2 | 2 | 1 | 1 | 1 | 0.1 | 2 | 0.004 | <0.01 | 0.03 | 26 | <0.01 |
| **85–23** | Okochiri | <0.01 | <0.01 | <0.01 | 2 | 1 | 1 | 1 | 1 | 0.1 | 5 | <0.001 | <0.01 | 0.02 | 29 | <0.01 |
| **86–22** | Okochiri | 1 | <0.01 | <0.01 | 1 | 1 | 1 | 1 | 0.2 | 0.1 | 1 | 0.002 | <0.01 | 0.02 | 26 | 1.4 |
| **87–22** | Okochiri | 3 | <0.01 | <0.01 | 2 | 2 | 0.4 | 2 | 0.2 | 0.1 | 1 | 0.002 | 0.02 | 0.02 | 29 | 1.5 |
| **88–22** | Okochiri | 1 | <0.01 | <0.01 | 2 | 1 | 1 | 1 | 0.2 | 0.1 | 1 | 0.004 | <0.01 | <0.02 | 29 | 0.5 |
| **88–23** | Okochiri | <0.01 | <0.01 | <0.01 | 2 | 3 | 1 | 1 | 0.1 | 0.1 | 4 | <0.001 | <0.01 | <0.02 | 30 | <0.01 |
| **89–22** | Okochiri | <0.01 | <0.01 | <0.01 | 2 | 6 | 1 | 2 | 0.2 | 0.2 | 1 | 0.003 | 25 | 0.1 | 31 | <0.01 |
| **ID** | **Site** | **NO₃ (mg/L)** | **NO₂ (mg/L)** | **F (mg/L)** | **Cl (mg/L)** | **SO₄² (mg/L)** | **Ca (mg/L)** | **Na (mg/L)** | **K (mg/L)** | **Mg (mg/L)** | **Si (mg/L)** | **Sr (mg/L)** | **Fe (mg/L)** | **Mn (mg/L)** | **DOC (mg/L)** | **NO₃^-^/Cl^-^** |
| **89–23** | Okochiri | <0.01 | <0.01 | <0.01 | 3 | 7 | 1 | 2 | 0.1 | 0.1 | 1 | <0.001 | 6 | 0.1 | 26 | <0.01 |
| **90–22** | Okochiri | 1.4 | <0.01 | <0.01 | 2 | 2 | 1 | 2 | <0.01 | 0.1 | 2 | 0.002 | 0.1 | 0.1 | 24 | 0.7 |
| **91–22** | Okochiri | 1 | <0.01 | <0.01 | 1 | 1 | 1 | 1 | 0.2 | 0.1 | 1 | 0.003 | <0.01 | <0.02 | 28 | 1 |
| **92–22** | Okochiri | 1 | <0.01 | <0.01 | 1 | 1 | 1 | 1 | 1 | 0.2 | 7 | 0.01 | <0.01 | 0.02 | 21 | 1 |
| **93–22** | Okochiri | 2 | <0.01 | <0.01 | 2 | 2 | 1 | 1 | 0.3 | 0.2 | 2 | 0.002 | <0.01 | 0.04 | 31 | 1 |
| **94–22** | Okochiri | 2 | <0.01 | <0.01 | 2 | 2 | 0.4 | 1 | 1 | 0.1 | 2 | 0.002 | <0.01 | 0.03 | 42 | 1 |
| **94–23** | Okochiri | <0.01 | <0.01 | 1 | 2 | 1 | 0.3 | 1 | 0.4 | 0.1 | 5 | <0.001 | <0.01 | 0.03 | 26 | <0.01 |
| **95–22** | Okochiri | 2 | <0.01 | <0.01 | 2 | 2 | 1 | 1 | 0.4 | 0.2 | 1 | 0.003 | <0.01 | 0.04 | 18 | 1 |
| **96–22** | Okochiri | 2 | <0.01 | <0.01 | 2 | 1 | 1 | 1 | 1 | 0.1 | 1 | 0.004 | <0.01 | 0.03 | 22 | 1 |
| **97–22** | Okochiri | 2 | <0.01 | <0.01 | 1.4 | 2 | 0.4 | 1 | 0.4 | 0.2 | 1 | 0.001 | <0.01 | 0.04 | 25 | 1.4 |
| **98–22** | Okochiri | 2 | <0.01 | <0.01 | 2 | 1 | 1 | 1 | 0.3 | 0.1 | 1 | 0.003 | <0.01 | <0.02 | 18 | 1 |
| **99–22** | Okochiri | 2 | <0.01 | <0.01 | 1 | 2 | 1 | 1 | 0.3 | 0.1 | 1 | 0.004 | <0.01 | 0.02 | 14 | 2 |
| **100–22** | Okochiri | 10 | <0.01 | 0.1 | 2 | 4 | 4 | 2 | 0.4 | 0.3 | 1 | 0.01 | 0.04 | 0.04 | 43 | 5 |
| **101–22** | Okochiri | 2 | <0.01 | <0.01 | 2 | 3 | 1 | 1 | 0.3 | 0.2 | 1 | 0.003 | <0.01 | 0.1 | 29 | 1 |
| **102–22** | Okochiri | 1 | <0.01 | <0.01 | 2 | 2 | 1 | 1 | 0.4 | 0.2 | 1 | 0.001 | 0.02 | 0.02 | 32 | 0.5 |
| **103–22** | Okochiri | 2 | <0.01 | <0.01 | 2 | 2 | 2 | 1 | 0.2 | 0.4 | 1 | 0.01 | <0.02 | <0.02 | 30 | 1 |
| **104–22** | Okochiri | 2 | <0.01 | <0.01 | 2 | 2 | 1 | 1 | 0.2 | 0.1 | 1 | 0.003 | 0.02 | <0.02 | 37 | 1 |
| **105–22** | Okochiri | <0.01 | <0.01 | <0.01 | 2 | 2 | 1 | 1 | 0.4 | 0.2 | 1 | 0.002 | <0.01 | 0.02 | 23 | <0.01 |
| **106–22** | Okochiri | 2 | <0.01 | <0.01 | 2 | 6 | 2 | 1 | 0.4 | 0.3 | 1 | 0.01 | <0.01 | 0.1 | 29 | 1 |
| **107–22** | Okochiri | 2 | <0.01 | <0.01 | 1 | 2 | 1 | 1 | 0.2 | 0.1 | 1 | 0.002 | <0.01 | 0.02 | 34 | 2 |
| **108–22** | Okochiri | 2 | <0.01 | <0.01 | 1 | 2 | 0.4 | 3 | 0.2 | 0.1 | 1 | 0.001 | <0.01 | 0.02 | 30 | 2 |
| **108–23** | Okochiri | <0.01 | <0.01 | <0.01 | 2 | 1 | 0.4 | 4 | 0.2 | 0.1 | 5 | 0.001 | <0.01 | <0.02 | 29 | <0.01 |
| **109–23** | Okochiri | 1 | <0.01 | <0.01 | 2 | 1 | 1 | 1 | 0.2 | 0.1 | 4 | <0.001 | <0.01 | 0.03 | ND | 0.5 |
| **110–23** | Okochiri | 2 | <0.01 | <0.01 | 3 | 3 | 1 | 3 | 0.3 | 0.2 | 5 | <0.001 | 0.03 | 0.04 | ND | 0.7 |
| **111–23** | Okochiri | 3 | <0.01 | <0.01 | 5 | 7 | 1 | 2 | 0.2 | 0.1 | 4 | <0.001 | <0.01 | 0.02 | ND | 0.6 |
| **112–23** | Okochiri | 1 | <0.01 | <0.01 | 2 | 2 | 0.4 | 1.4 | 0.3 | 0.1 | 5 | <0.001 | <0.01 | 0.03 | ND | 0.5 |
| **113–23** | Okochiri | 1 | <0.01 | <0.01 | 3 | 12 | 1 | 3 | 0.1 | 0.2 | 1 | <0.001 | 14 | 0.1 | ND | 0.3 |
| **114–23** | Okochiri | <0.01 | <0.01 | <0.01 | 4 | 2 | 0.2 | 2 | 0.1 | < 0.01 | 3 | <0.001 | 2 | 0.01 | 29 | <0.01 |
| **115–23** | Okochiri | <0.01 | <0.01 | 1 | 5 | 6 | 0.3 | 1.4 | 0.1 | 0.1 | 3 | <0.001 | 1 | 0.04 | 31 | <0.01 |
| **Minimum** | | <0.01 | <0.01 | <0.01 | 1 | 1 | 0.2 | 1 | <0.01 | <0.01 | 1 | <0.001 | <0.01 | <0.02 | 2 | <0.01 |
| **Maximum** | | 10 | 0.2 | 1.3 | 5 | 12 | 4 | 15 | 1 | 0.4 | 7 | 0.01 | 25 | 1 | 43 | 5 |
| **Median** | | 2 | <0.01 | 1 | 2 | 2 | 1 | 2 | 0.3 | 0.2 | 2 | 0.003 | 0.1 | 0.04 | 26 | 1 |
| **Average** | | 2 | <0.01 | 0.8 | 2 | 3 | 1 | 3 | 0.4 | 0.2 | 2 | 0.004 | 3 | 0.1 | 25 | 1 |
| **All data** | | | | | | | | | | | | | | | | |
| **Minimum** | | <0.01 | <0.01 | <0.01 | 1 | <0.01 | 0.2 | 1 | 0.1 | <0.01 | 0.3 | <0.001 | <0.01 | <0.02 | 2 | <0.01 |
| **Maximum** | | 211 | 2 | 3 | 66 | 80 | 51 | 56 | 59 | 14 | 8 | 1 | 50 | 2 | 54 | 5 |
| **Median** | | 3 | 0.2 | 0.2 | 3 | 2 | 1 | 3 | 0.4 | 0.2 | 1 | 0.01 | 0.2 | 0.04 | 28 | 1.4 |
| **Average** | | 25 | 1 | 0.4 | 8 | 6 | 5 | 8 | 3 | 1 | 2 | 0.03 | 4 | 0.2 | 27 | 2 |
| **REF1** | Alode | 3 | <0.01 | <0.01 | 5 | 6 | 0.3 | 2 | 0.1 | 0.1 | 3 | <0.001 | <0.01 | <0.01 | 23 | 0.6 |
| **REF2** | Alode | 3 | <0.01 | <0.01 | 5 | 6 | 1 | 1 | 0.1 | 0.1 | 2 | <0.001 | <0.01 | <0.01 | 35 | 0.6 |
| **REF3** | Okochiri | <0.01 | <0.01 | <0.01 | 4 | 6 | 1 | 1 | 0.3 | 0.1 | 3 | <0.001 | <0.01 | <0.01 | 2 | <0.01 |
| **REF4** | Okrika | 2 | <0.01 | <0.01 | 2 | 2 | 1 | 1 | 0.6 | 0.3 | 2 | 0.004 | <0.01 | 0.03 | 10 | 1 |
| **REF5** | Ogale | 1 | <0.01 | <0.01 | 2 | 2 | 1 | 0.4 | 0.3 | 0.1 | 1 | 0.001 | <0.01 | 0.02 | 22 | 0.7 |

*Notes: ND = Not Determined, DOC = Dissolved Organic Carbon, REF = Reference sample. Sample names that end with –22 and –23 represent groundwater samples collected in 2022 and 2023, respectively.*

**Table 3S: Chemical measurements of sewage in the study area**

| **Sample Name** | **NO₃ (mg/L)** | **NO₂ (mg/L)** | **Cl (mg/L)** | **F (mg/L)** | **PO₄^3^ (mg/L)** | **SO₄² (mg/L)** | **Ca (mg/L)** | **Na (mg/L)** | **K (mg/L)** | **Mg (mg/L)** | **Si (mg/L)** | **Sr (mg/L)** | **Fe (mg/L)** | **Mn (mg/L)** |
| --- | --- | --- | --- | --- | --- | --- | --- | --- | --- | --- | --- | --- | --- | --- |
| **EF1** | 127 | <0.01 | 45 | 0.3 | <0.01 | 21 | 30 | 36 | 19 | 5 | 2 | 0.1 | 0.4 | 0.1 |
| **EF2** | 131 | 0.4 | 99 | <0.01 | 10 | 8 | 39 | 5 | 1 | 2 | 5 | 0.3 | 1 | 0.2 |
| **EF3** | <0.01 | <0.01 | 248 | <0.01 | 54 | 2 | 37 | 363 | 74 | 13 | 31 | 0.2 | 7 | 0.3 |
| **EF4** | 103 | <0.01 | 43 | <0.01 | <0.01 | 20 | 60 | 65 | 50 | 12 | 6 | 0.3 | 3 | 1 |
| **EF5** | 145 | <0.01 | 123 | 0.1 | 2 | 10 | 65 | 83 | 40 | 7 | 8 | 0.4 | 1 | 1 |
| **EF6** | 1 | <0.01 | 68 | 0.1 | <0.01 | 6 | 39 | 28 | 16 | 1 | 5 | 0.3 | 0.3 | 0.2 |
| **EF7** | 31 | <0.01 | 26 | <0.01 | 2 | 9 | 22 | 23 | 10 | 2 | 3 | 0.1 | 0.1 | 0.2 |
| **EF8** | 42 | <0.01 | 31 | 0.1 | 2 | 6 | 34 | 30 | 13 | 3 | 5 | 0.2 | 0.3 | 0.2 |
| **Minimum** | <0.01 | <0.01 | 26 | <0.01 | <0.01 | 2 | 22 | 5 | 1 | 1 | 2 | 0.1 | 0.1 | 0.1 |
| **Maximum** | 145 | 0.4 | 248 | 0.3 | 54 | 21 | 65 | 363 | 74 | 13 | 31 | 0.4 | 7 | 1 |
| **Median** | 103 | <0.01 | 57 | 0.1 | 2.3 | 8.5 | 38 | 33 | 17.5 | 4 | 5 | 0.3 | 0.7 | 0.2 |
| **Average** | 83 | <0.01 | 85 | 0.2 | 14 | 10 | 41 | 79 | 28 | 6 | 8 | 0.2 | 2 | 0.4 |
| **REF1** | <0.01 | <0.01 | 6 | 0.1 | <0.01 | 1 | 18 | 12 | 5 | 1 | 3 | 0.1 | 0.1 | 0.0 |
| **REF2** | 1 | <0.01 | 12 | <0.01 | <0.01 | 4 | 65 | 37 | 44 | 8 | 8 | 0.3 | 8 | 1 |


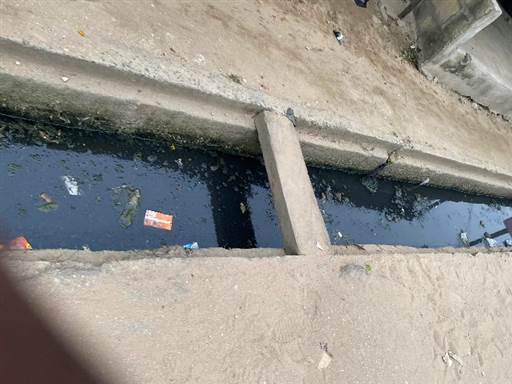

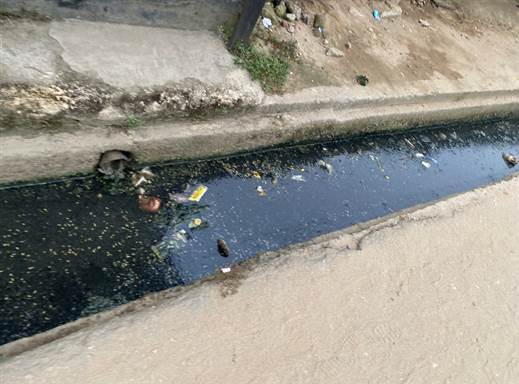


B

A


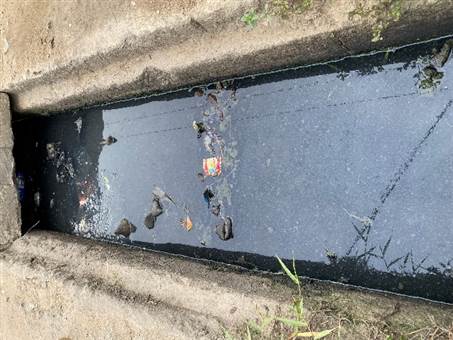

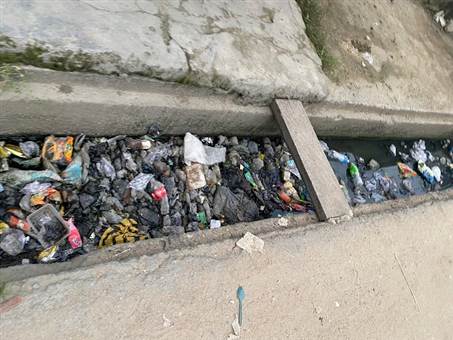

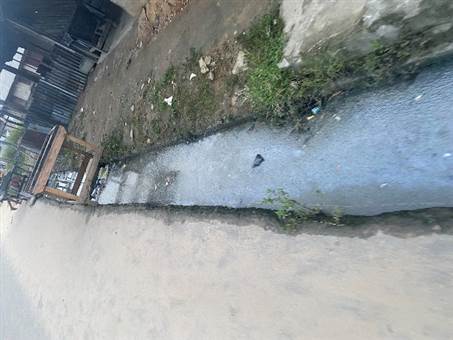


E

D

C


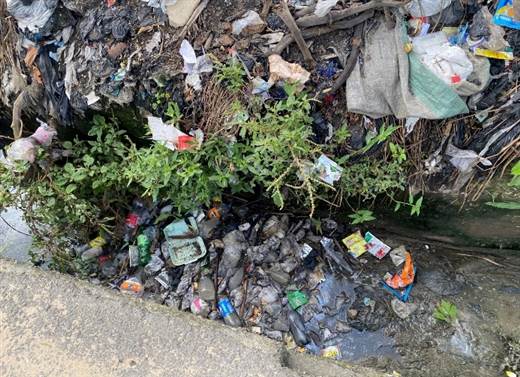

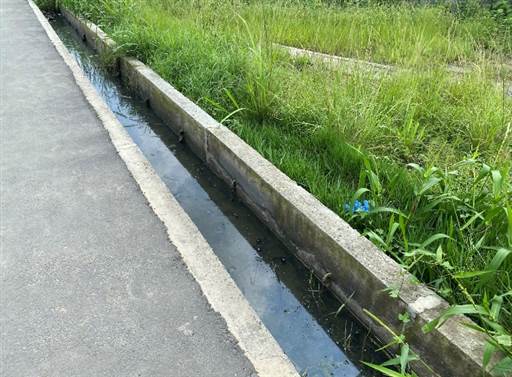


G

F

**Fig. S1.** (A), (B), (C), (D), and (E) municipal sewage in the Alesa, and (F) municipal sewage in the Ogale, and (G) municipal sewage in the Ebubu drainage systems. The arrow shows the flow direction.


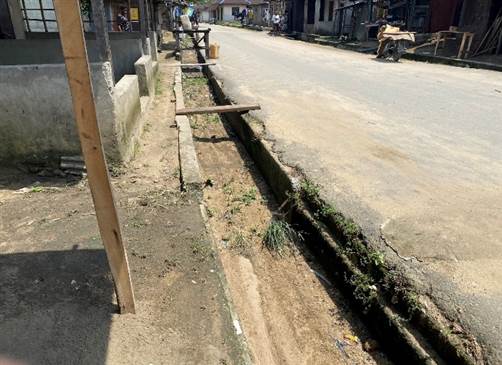

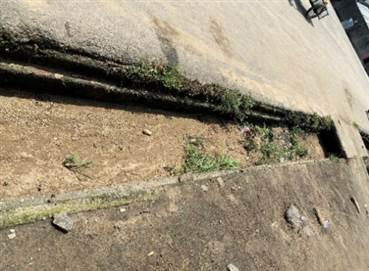

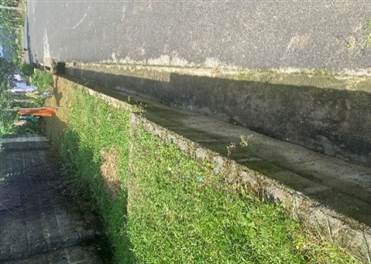


**Fig. S2.** Municipal drainage systems in Okochiri. Municipal sewage was not ansent. The arrow shows the flow direction.


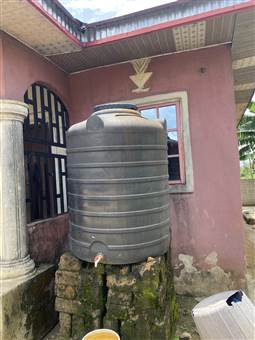

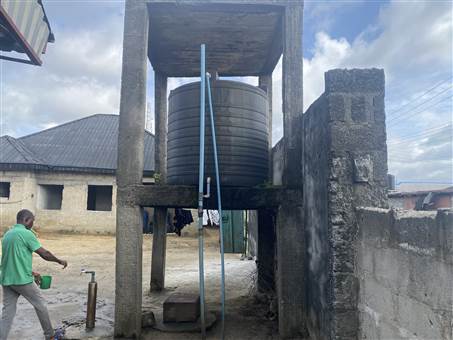

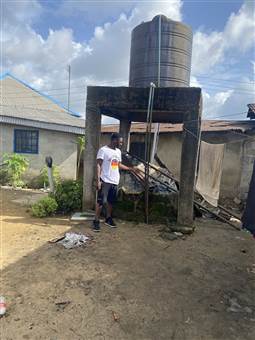


B

C

A


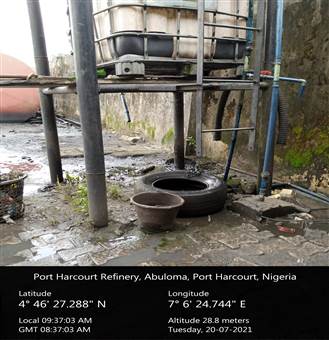

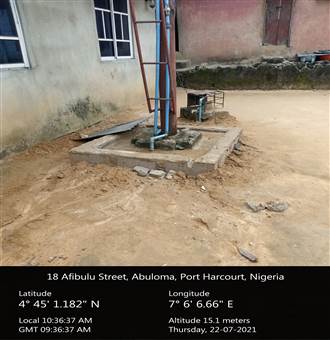

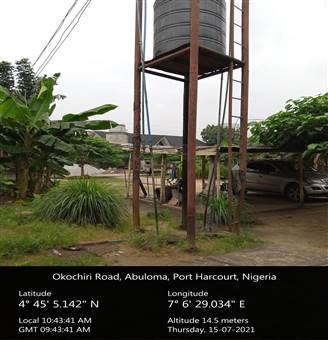


E

F

D

**Wellhead**

**Wellhead**

**Wellhead**

**Fig. S3.** The overhead storage tank for drinking water in the (A), (D), and (E) Okochiri, (B), (C), and (F) Ogale. Wellheads sealed with concrete slaps in Okochiri (D and E) and Ogale (F).


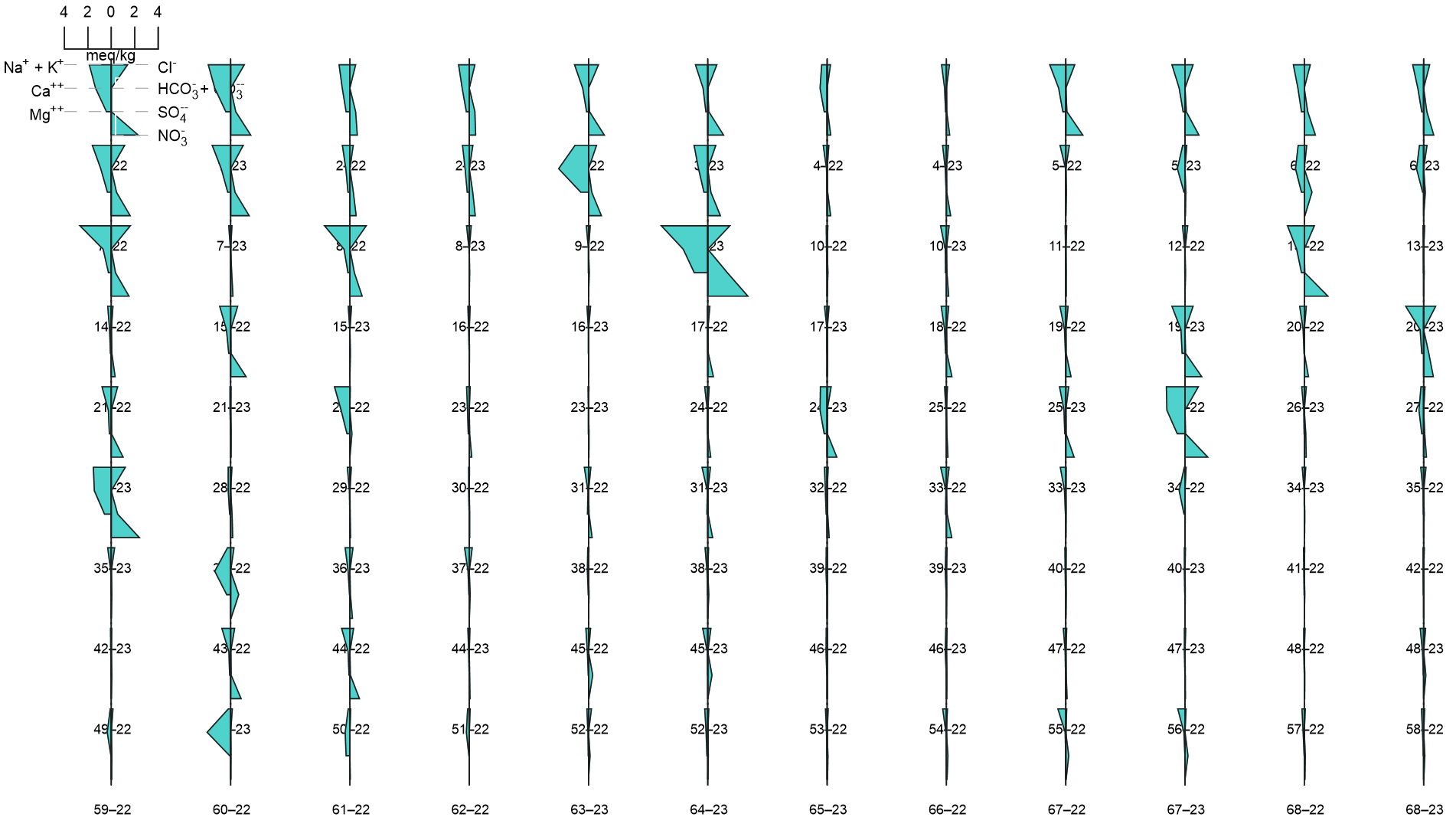


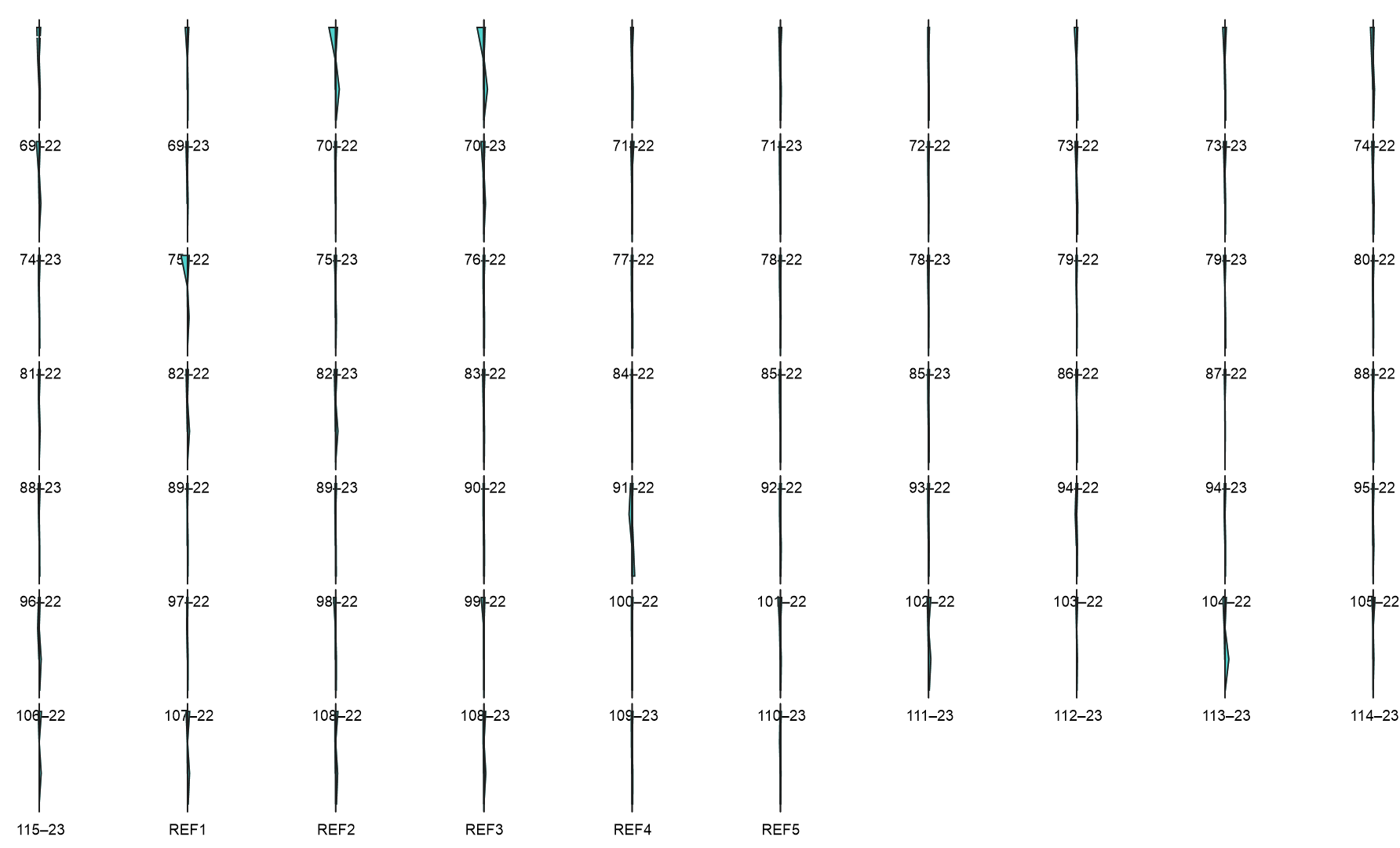


**Fig. S4.** Stiff diagrams of groundwater samples in the study area.


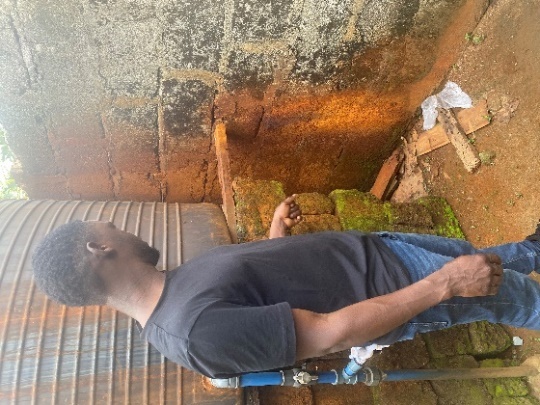

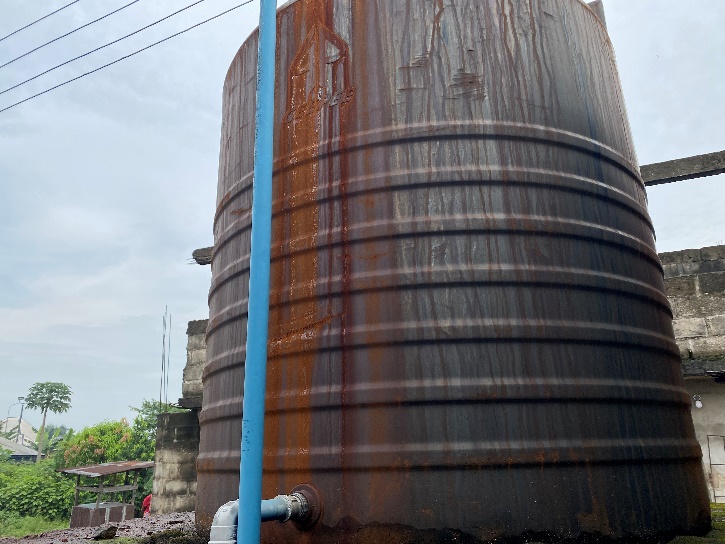


D

C

B

A


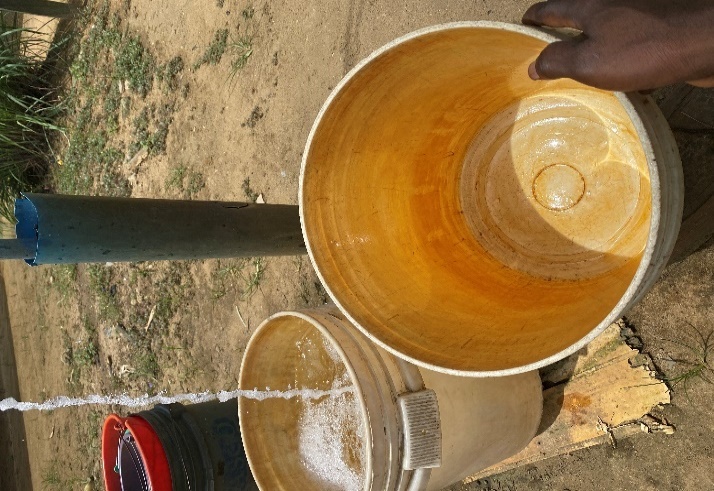

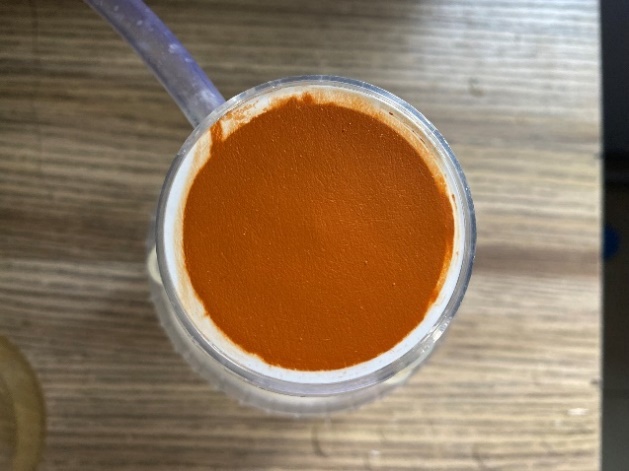


**Fig. S5** (A, B and C) Reddish-brown rust particles on **polyvinyl chloride** overhead water storage tanks and other domestic water containers and (D) rust particles on 0.45 μm cellulose acetate membrane filters due to iron precipitation.in Ogale groundwater.
